# The interferon-inducible protein viperin controls cancer metabolic reprogramming to enhance cancer progression

**DOI:** 10.1172/JCI157302

**Published:** 2022-12-15

**Authors:** Kyung Mi Choi, Jeong Jin Kim, Jihye Yoo, Ku Sul Kim, Youngeun Gu, John Eom, Haengdueng Jeong, Kyungeun Kim, Ki Taek Nam, Young Soo Park, Joon-Yong Chung, Jun-Young Seo

**Affiliations:** 1 Severance Biomedical Science Institute, Graduate School of Medical Science, Brain Korea 21 Project, Yonsei University College of Medicine, Seoul, South Korea.; 2 Department of Pathology, Kangbuk Samsung Hospital, Sungkyunkwan University School of Medicine, Seoul, South Korea.; 3 Department of Pathology, Asan Medical Center, University of Ulsan College of Medicine, Seoul, South Korea.; 4 Molecular Imaging Branch, Center for Cancer Research, National Cancer Institute (NCI), NIH, Bethesda, Maryland, USA.

**Keywords:** Metabolism, Oncology, Cancer, Fatty acid oxidation, Glucose metabolism

## Abstract

Metabolic reprogramming is an important cancer hallmark. However, the mechanisms driving metabolic phenotypes of cancer cells are unclear. Here, we show that the interferon-inducible (IFN-inducible) protein viperin drove metabolic alteration in cancer cells. Viperin expression was observed in various types of cancer and was inversely correlated with the survival rates of patients with gastric, lung, breast, renal, pancreatic, or brain cancer. By generating viperin knockdown or stably expressing cancer cells, we showed that viperin, but not a mutant lacking its iron-sulfur cluster–binding motif, increased lipogenesis and glycolysis via inhibition of fatty acid β-oxidation in cancer cells. In the tumor microenvironment, deficiency of fatty acids and oxygen as well as production of IFNs upregulated viperin expression via the PI3K/AKT/mTOR/HIF-1α and JAK/STAT pathways. Moreover, viperin was primarily expressed in cancer stem-like cells (CSCs) and functioned to promote metabolic reprogramming and enhance CSC properties, thereby facilitating tumor growth in xenograft mouse models. Collectively, our data indicate that viperin-mediated metabolic alteration drives the metabolic phenotype and progression of cancer.

## Graphical abstract

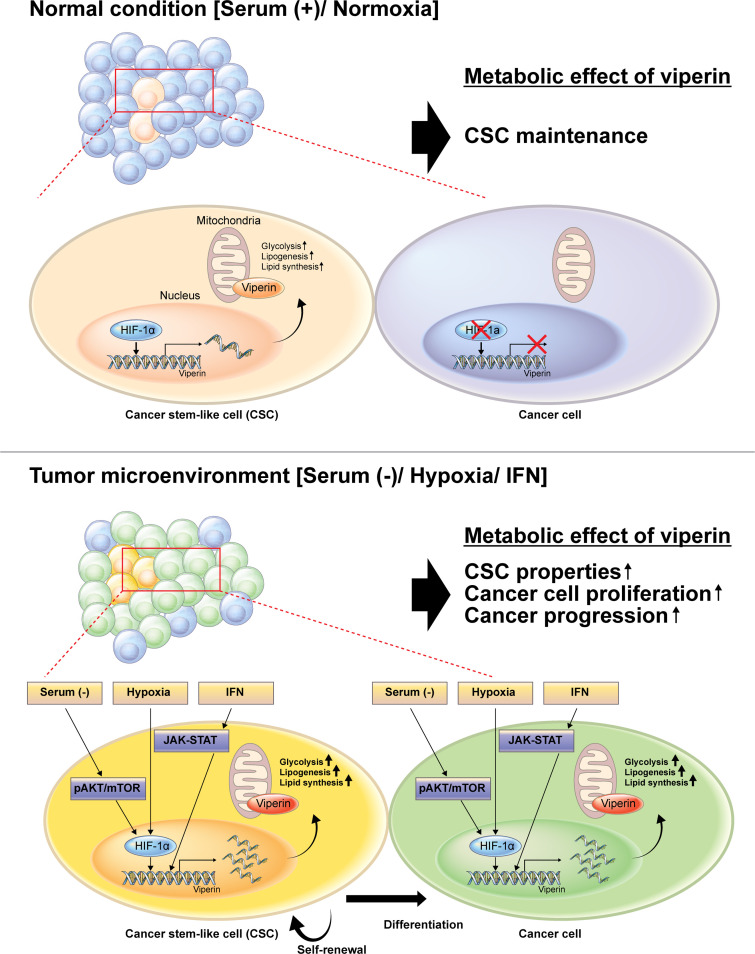

## Introduction

Cancers are surrounded by a complex microenvironment that causes hypoxic and nutritional stress. Cancer cells reprogram metabolic pathways to support cancer proliferation, growth, metastasis, and survival within this harsh condition (1, 2). Increases in glycolysis, glutaminolysis, mitochondrial biogenesis, and lipid metabolism are among the most prominent metabolic alterations in cancer (3, 4). These processes provide not only energy to cancer cells but also essential factors to support their biosynthesis and proliferation (5, 6).

The PI3K/AKT pathway is a canonical pathway mediating the regulation of cancer metabolism (7). PI3K/AKT signaling activates mTOR, which activates the transcription factor HIF-1. HIF-1 induces the expression of glucose transporters and glycolytic enzymes (8, 9) and negatively regulates mitochondrial biogenesis and oxygen consumption (10, 11). The PI3K/AKT pathway also contributes to the enhancement of lipogenesis, which is associated with cancer progression and metastasis (12, 13). This pathway stimulates synthesis of the transcription factor SREBP-1 as well as nuclear accumulation of its active form, resulting in an increase of cellular fatty acids and phospholipids. Nevertheless, PI3K/AKT signaling only partially accounts for our understanding of the mechanisms driving metabolic phenotypes of cancer cells.

Interferons (IFNs) are a family of pleotropic cytokines that induce an anticancer immune response. Emerging evidence suggests that IFNs also play an important role in cancer metabolism (14, 15). Specifically, IFNs activate JAK/STAT signaling, which regulates the metabolic process (15, 16). Additionally, IFN regulatory factors (IRFs) are involved in the modulation of metabolism in cancer (17–19). IFNs upregulate the transcription of a large number of IFN-stimulated genes (ISGs), whose products play a major role in the immune response (20). However, the role of ISGs in cancer metabolism is poorly understood and not widely studied.

Viperin (also known as RSAD2, cig5, or vig1) is an ISG-encoded protein. It has multiple functions in various cell types including fibroblasts, adipocytes, and macrophages (21–24). Viperin exhibits antiviral activity against a wide variety of viruses, mediates signaling pathways, and modulates cellular metabolism (25). This protein inhibits fatty acid β-oxidation in mitochondria, which in turn reduces ATP generation and enhances glycolysis and lipogenesis during human CMV infection (23, 24), suggesting that its function can be exploited to drive metabolic alteration of cancer cells.

In this study, we initially investigated the correlation between clinical outcomes and viperin expression in cancer tissues from patients with gastric, lung, or breast cancer. We then determined viperin expression and localization in various conditioned cancer cell lines and cancer stem-like cells (CSCs; also known as tumor-initiating cells) to elucidate its mechanism of induction. We also assessed viperin regulation of cancer metabolism and progression by generating cancer cell lines with viperin knockdown (KD) or stable expression of viperin and quantifying the expression levels of lipogenesis-related genes. Last, we examined whether viperin-mediated cancer metabolism affects the tumorigenesis capacity of CSCs in vivo by monitoring tumor growth in mice inoculated with CSCs.

## Results

### Viperin expression in human cancer tissues correlates with clinical outcomes.

**Figure 1:**
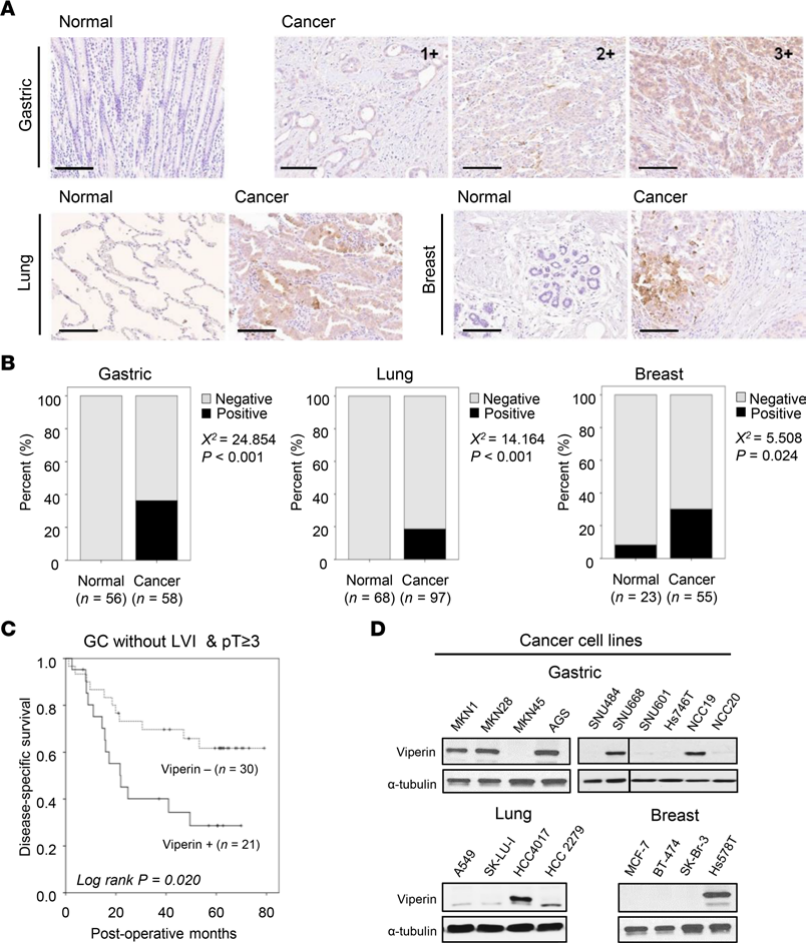
Viperin expression in human cancer tissues and its correlation with survival of patients with cancer. (**A** and **B**) Viperin was specifically expressed in cancer tissues. (**A**) IHC analysis of viperin expression in human gastric (*n* = 114), lung (*n* = 165), and breast cancer (*n* = 78) tissues stained with a mAb against viperin (MaP.VIP). The paired adjacent noncancerous tissues were used for comparison against cancer tissues. A score of 1 was considered to represent mild expression, 2 as moderate expression, and 3 as marked expression. Scale bars: 100 μm. (**B**) Statistical analysis of viperin expression in cancer specimens. Pearson’s χ^2^ test and *P* values are shown for cancerous versus normal tissues. (**C**) Disease-specific survival of patients with gastric cancer (GC). In a subgroup (*n* = 51) of combined advanced-stage (pT ≥3) without lymphovascular invasion (LVI), patients with high expression of viperin had poor disease-free survival (Kaplan-Meier plot). A log-rank test (*P* = 0.020) was performed for patients with high and low expression of viperin. (**D**) Viperin expression in various cancer cell lines. Viperin protein was detected by immunoblotting using MaP.VIP. α-Tubulin was used as a loading control.

To investigate whether viperin is expressed in human cancer tissues with clinical significance, we performed IHC on human gastric, lung, and breast cancer tissue microarrays. Viperin showed cytoplasmic positivity in cancer cells with variable intensity (Figure 1A and Supplemental Figure 1A; supplemental material available online with this article; https://doi.org/10.1172/JCI157302DS1). Viperin expression was significantly increased in cancer tissues compared with expression in their adjacent noncancerous tissue samples (Figure 1B). We next examined the relationship of viperin expression with the outcomes of patients. Patients with high expression of viperin in the advanced stage of gastric cancer showed a reduced survival trend compared with that of patients with low expression of viperin (Supplemental Figure 1B). In a subgroup of patients in the advanced stage without lymphovascular invasion, those with high expression of viperin showed worse disease-free survival than did patients with low expression of viperin (mean of 29.31 vs. 45.63 months, *P* = 0.020) (Figure 1C). We also analyzed published data sets for patients with various types of cancer such as gastric adenocarcinoma (26), lung adenocarcinoma (27), breast cancer (28), renal cell carcinoma (29), pancreatic adenocarcinoma (30), and glioblastoma (31) to determine the relationship between viperin expression and the outcomes of the patients. Similar to our data set, the expression level of viperin was inversely proportional to the survival rate of patients with cancer (*P* < 0.05; Supplemental Figure 1C). The data indicate that viperin is highly expressed in cancer tissues, which associates with adverse clinical outcomes.

To examine the role of viperin in cancer, we initially screened viperin expression in a variety of cancer cell lines. Viperin protein expression was detected in certain cell lines from gastric cancer (MKN1, MKN28, AGS, SNU668, and NCC19), lung cancer (HCC4017), and breast cancer (Hs578T) by immunoblotting (Figure 1D) and confocal immunofluorescence microscopy (Supplemental Figure 1D). Quantitative reverse transcription PCR (qRT-PCR) revealed similar findings for viperin gene expression (Supplemental Figure 1E). The results indicate that viperin is not expressed by default in all cancer tissues and cell lines. We, therefore, selected several specific cancer cell lines for further experiments.

### Viperin regulates fatty acid and glucose metabolism of cancer.

**Figure 2:**
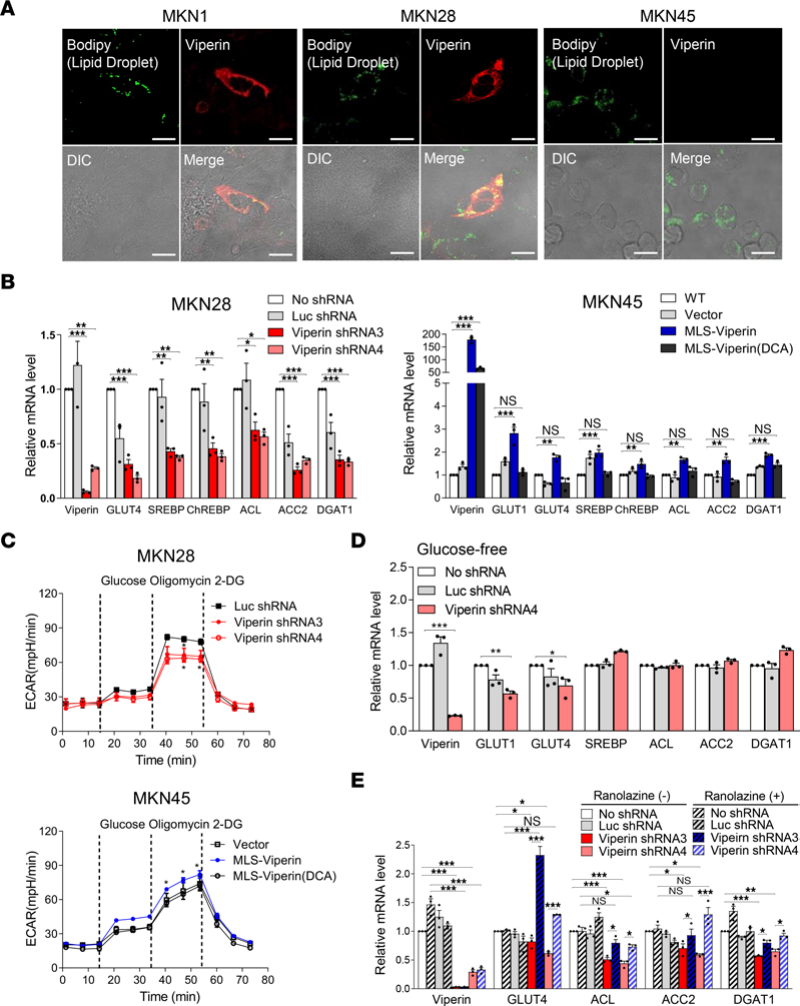
Viperin induces lipogenesis and glycolysis of cancer cells. (**A**) Lipid accumulation in cancer cells expressing viperin. Gastric cancer cell lines expressing viperin (MKN1 and MKN28) and those not expressing viperin (MKN45) were stained with bodipy-FITC (green), an indicator of LDs, and MaP.VIP (red). Scale bars: 20 μm. (**B**) No shRNA, a control Luc shRNA, or viperin shRNAs were stably expressed in MKN28 cells, and a control vector, MLS-viperin, or MLS-viperin (DCA) was stably expressed in MKN45 cells. Relative mRNA levels of viperin, glucose transporters (GLUT1 and -4), major transcriptional regulators (SREBP and ChREBP), and key lipogenic enzymes (ACL, ACC2, and DGAT1) in the stable cell lines were measured by qRT-PCR and normalized to *ACTB* mRNA. Data are presented as the mean ± SEM (*n* = 3 in triplicate). MLS-viperin, the N-terminal amphipathic α-helix (residues 1 to 42) of viperin was deleted and replaced by MLSs (residues 2 to 34) of vMIA; MLS-Viperin (DCA), 2 cysteine residues (88 and 91) of MLS-viperin were mutated to alanine. (**C**) The ECAR was measured in the MKN28 and MKN45 stable cell lines. Glucose, oligomycin, and 2-deoxyglucose (2-DG) were added at the indicated time points. Data are presented as the mean ± SEM (*n* = 3 in triplicate). (**D** and **E**) MKN28 viperin-KD cells were incubated in glucose-free media (**D**) or treated with ranolazine (**E**) for 24 hours. Relative mRNA levels of the indicated genes were measured. Data are presented as the mean ± SEM (*n* = 2 in triplicate). *P < 0.05, **P < 0.01, and ***P < 0.001, by 1-way ANOVA with Dunnett’s multiple-comparison test (**B**, **C**, and **D**) or Tukey’s multiple-comparison test (**E**).

Viperin plays multiple roles depending on its intracellular localization in various cell types (25). We monitored the intracellular distribution of viperin in gastric cancer tissues and cells by immunofluorescence microscopy (Supplemental Figure 2). Consistent with the IHC results, we detected viperin in the cytoplasm of cancer cells with variable intensity, and its expression was significantly increased in cancer tissues compared with levels in normal tissues (Supplemental Figure 2A). Moreover, viperin was observed in mitochondria of cancer tissue samples (Supplemental Figure 2, B and C) and the cancer cell line MKN28 (Supplemental Figure 2D). The mitochondrial localization of viperin suggests that it can potentially alter metabolism in cancer cells. To test this, we analyzed the formation of lipid droplets (LDs), which act as storage compartments for triglycerides and long chain fatty acids in cancer cell lines (32). Viperin-expressing cells showed intense accumulation of LDs, whereas nonexpressing cells had only basal levels of LDs, which varied for each cancer cell line (Figure 2A and Supplemental Figure 2E). This suggests that viperin expression affects the fatty acid metabolism of cancer cells. To assess whether viperin regulates cancer metabolism, we generated cancer cell lines with viperin KD or stable expression of viperin. Viperin expression in MKN1, MKN28, and AGS was suppressed by stable expression of 4 different viperin shRNAs. The viperin-KD efficiency was over 90%, as shown by immunoblotting (Supplemental Figure 3A) and qRT-PCR (Supplemental Figure 3B) compared with control cells expressing no shRNA (WT) or luciferase shRNA (Luc shRNA, control). Meanwhile, WT viperin and 3 mutants — viperin (DCA), in which 2 cysteine residues (88 and 91) of viperin were mutated to alanine to eliminate Fe-S cluster association (33); MLS-viperin, in which the N-terminal amphipathic α-helix (residues 1 to 42) of viperin were deleted and replaced by mitochondrial localization sequences (MLSs) for trafficking to mitochondria (24); and MLS-viperin (DCA), in which 2 cysteine residues (88 and 91) of MLS-viperin were mutated to alanine — were stably expressed in MKN45 cells. The expression levels of all constructs were confirmed by immunoblotting and qRT-PCR (Supplemental Figure 3, A and B). Viperin-KD cells showed slower proliferation compared with WT and control cells (Supplemental Figure 3C), suggesting that viperin expression affected the proliferation of cancer cells. We also measured expression levels of the glucose transporters GLUT1 and GLUT4; the major transcriptional regulators sterol regulatory element–binding protein (SREBP) and carbohydrate–responsive element–binding protein (ChREBP); and the key lipogenic enzymes ATP-citrate lyase (ACL), acetyl-CoA carboxylase (ACC) 2, and diacylglycerol acyltransferase 1 (DGAT1). The expression levels of these genes were significantly reduced in viperin-KD cells compared with expression levels in MKN28, MKN1, and AGS control cells (Figure 2B and Supplemental Figure 4A). Their expression levels were increased in cells stably expressing WT viperin or MLS-viperin, but not in cells expressing viperin (DCA) or MLS-viperin (DCA) compared with MKN45 controls (Figure 2B and Supplemental Figure 4A). The LD formation was also substantially reduced in viperin-KD cells (Supplemental Figure 4B). Inversely, the levels of fatty acid β-oxidation were significantly increased in viperin-KD cells compared with MKN28 control cells (Supplemental Figure 4C). In addition, the oxygen consumption rate (OCR), an indicator of mitochondrial respiration, was increased in viperin-KD cells compared with MKN28 control cells and was reduced in cells stably expressing MLS-viperin, but not in cells expressing MLS-viperin (DCA) compared with MKN45 controls (Supplemental Figure 4D). Next, to assess whether viperin affects glucose metabolism of these cancer cell lines, we analyzed the extracellular acidification rate (ECAR), an indicator of aerobic glycolysis (Figure 2C and Supplemental Figure 4E). As expected, the glycolytic capacity was reduced in viperin-KD cells compared with MKN28 controls (Figure 2C) and was increased in cells expressing WT viperin and in those stably expressing MLS-viperin, but not in cells expressing viperin (DCA) or MLS-viperin (DCA) compared with MKN45 controls (Figure 2C and Supplemental Figure 4E). Meanwhile, the cellular ATP levels were reduced in WT viperin and cells stably expressing MLS-viperin, but not in cells expressing viperin (DCA) or MLS-viperin (DCA) compared with MKN45 controls (Supplemental Figure 4F). These findings indicate that viperin expression promotes lipogenesis and glycolysis in cancer cells and that the Fe-S binding motif is necessary for its function. Viperin interaction with mitochondrial trifunctional protein (TFP) inhibits fatty acid β-oxidation, resulting in enhancement of glycolysis and lipogenesis in fibroblasts (23, 24). To confirm the mechanism of viperin-mediated cancer metabolism, we measured the expression levels of lipogenesis-related genes in MKN28 viperin-KD cells in glucose-free media (Figure 2D). Under these conditions, the expression levels of GLUT1 and GLUT4 were significantly reduced, but the levels of SREBP, ChREBP, and the lipogenic enzymes remained unchanged. The data support the existence of a mechanism by which viperin enhances GLUT expression to increase glucose uptake, which activates SREBP and ChREBP to promote lipogenesis. We also examined the expression levels of lipogenesis-related genes in MKN28 viperin-KD cells treated with ranolazine, an inhibitor of TFP (Figure 2E). The expression levels of GLUT4 and lipogenic enzymes were restored in the ranolazine-treated viperin-KD cells. The results indicate that the inhibition of fatty acid β-oxidation by viperin interaction with TFP is required for lipogenesis in cancer cells. Taken together, our data indicate that viperin is a major driver of cancer metabolic reprogramming and that the Fe-S cluster binding motif of viperin is necessary for its function, suggesting a potential target for the development of anticancer therapeutics.

### Viperin is induced in the tumor microenvironment via PI3K/AKT/mTOR/HIF-1α and IFN signaling pathways to promote cancer metabolism.

**Figure 3:**
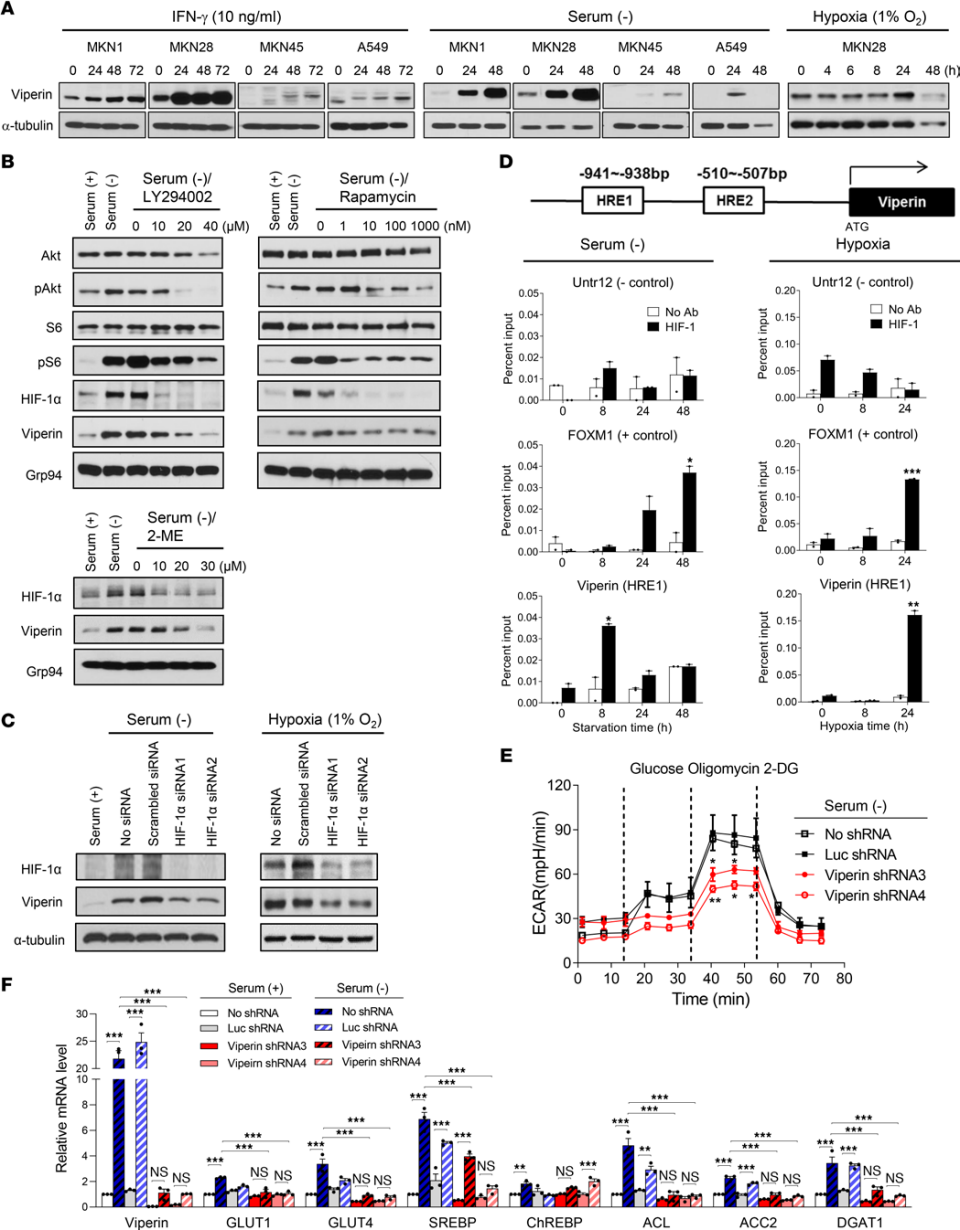
The mechanism of viperin induction in the TME. (**A**) Viperin induction in cancer cell lines under various culture conditions. Cells were treated with IFN-γ, cultured in serum-free media, or incubated in a hypoxia chamber for the indicated durations. Viperin was detected by immunoblot using MaP.VIP. α-Tubulin was used as a loading control. (**B**) MKN28 cells were cultured in the presence and absence of serum and treated with LY294002 (a PI3K/AKT inhibitor), rapamycin (an mTOR inhibitor), or 2-ME (an HIF-1α inhibitor) at the indicated concentration for 48 hours. Each protein was detected by immunoblot using specific mAbs. Grp94 was used as a loading control. (**C**) MKN28 cells were transfected with control or HIF-1α siRNAs and then cultured in the presence and absence of serum for 48 hours or incubated in a hypoxia chamber for 24 hours. Each protein was detected by immunoblot using specific mAbs. α-Tubulin was used as a loading control. (**D**) A ChIP assay was performed for MKN28 cells cultured in serum-free media or incubated in a hypoxia chamber for the indicated durations. A schematic representation of the HRE-binding sites in the viperin promoter region is shown. Chromatin samples were immunoprecipitated with a specific mAb against HIF-1 and assessed by real-time PCR. FOXM1 was used as a positive control and Untr12 as a negative control. Data are presented as the mean ± SEM (*n* = 2 in triplicate). (**E** and **F**) The ECAR (**E**) and lipogenesis levels (**F**) were measured in MKN28 stable cell lines cultured in serum-free media for 48 hours. Glucose, oligomycin, and 2-DG were added at the indicated time points (**E**). Relative mRNA levels of the indicated genes were measured by qRT-PCR and normalized to *ACTB* mRNA (**F**). Data are presented as the mean ± SEM (*n* = 2 in triplicate). *P < 0.05, **P < 0.01, and ***P < 0.001, by Student’s *t* test (**D**) and 1-way ANOVA with Dunnett’s multiple-comparison test (**E**) or Tukey’s multiple-comparison test (**F**).

In solid cancers, cancer cells reside in a nutrient- and oxygen-poor environment (34) and under the influence of cytokines including IFN-γ produced by immune cells (35). Consequently, cancer cells have to adapt their metabolism to proliferate and survive in this tumor microenvironment (TME) (36). To investigate whether viperin as a driver of cancer metabolic reprogramming is induced in the TME, we measured its expression levels under IFN-γ treatment, serum starvation, or hypoxia in various cancer cell lines (Figure 3A and Supplemental Figure 5A). As expected, viperin expression was increased in MKN1 and MKN28 cells but not in viperin-KD cells treated with IFN-γ. Upon IFN-γ treatment, viperin was also induced in cells without baseline expression, such as MKN45 and A549 cells. Moreover, we observed viperin induction under serum starvation and hypoxia, indicating that viperin was induced in the TME. Next, we examined the mechanism by which viperin is induced under these conditions. It is well known that viperin as an IFN-inducible protein is induced via the IFN signaling pathway under IFN-γ treatment (20). To confirm this, we treated MKN28 cells with the STAT3 inhibitor S31-201 under IFN-γ treatment (Supplemental Figure 5B). The induction levels of phosphorylated STAT3 (p-STAT3) and viperin were dose-dependently suppressed by the inhibitor under IFN-γ treatment. Many oncogenes and tumor suppressors are regulated via the PI3K/AKT/mTOR signaling pathway in various cancers (37, 38), and aberrant activation of this pathway allows cancer cells to achieve high levels of signaling with minimal dependence on extrinsic factors (39). The PI3K/AKT/mTOR pathway also activates HIF-1α, which is crucial for metabolic adaptation to hypoxia (40, 41). To assess whether viperin induction is regulated via the PI3K/AKT/mTOR/HIF-1α signaling pathway under serum starvation or hypoxia, we treated MKN28 cells with pathway-specific inhibitors or siRNAs (Figure 3, B and C, and Supplemental Figure 5C). We observed that the expression levels of p-AKT, p-S6 (ribosomal protein as a downstream effector of mTOR), HIF-1α, and viperin were increased in MKN28 under either serum starvation (Figure 3B) or hypoxia (Supplemental Figure 5C). When p-AKT activation was dose-dependently suppressed by the PI3K/AKT inhibitor LY294002, the expression levels of p-S6 and HIF-1α as downstream proteins of p-AKT were decreased along with the subsequent viperin expression levels under serum starvation (Figure 3B). When p-S6 activation was inhibited by the mTOR inhibitor rapamycin, the expression level of HIF-1α as a downstream protein of mTOR was decreased along with viperin expression under serum starvation (Figure 3B). When HIF-1α expression was suppressed by the HIF-1α inhibitor 2-methoxyestradiol (2-ME) or HIF-1α–specific siRNAs, we observed that viperin expression was reduced under both serum starvation (Figure 3, B and C) and hypoxic (Figure 3C and Supplemental Figure 5C) conditions. It is well known that PTEN negatively regulates the PI3K/AKT signaling pathway (42), and it has been reported that PTEN-KO mice have increased viperin expression levels (43). Thus, we also assessed the effect of PTEN on the mechanism of viperin induction under serum starvation (Supplemental Figure 5D). As expected, the level of PTEN expression was not affected by the PI3K/AKT inhibitor LY294002. However, when MKN28 cells were treated with the PTEN inhibitor SF1670 under serum starvation, the level of viperin expression was increased in a dose-dependent manner. The results indicate that viperin was induced via PI3K/AKT/mTOR/HIF-1α and IFN signaling pathways in the TME. To determine whether HIF-1α directly binds to the viperin promoter to regulate viperin expression, we performed a ChIP assay on MKN28 cells under either serum starvation or hypoxia (Figure 3D). Sequence analysis of the viperin promoter revealed 2 potential regions as hypoxia response elements (HREs). The ChIP assay showed that HIF-1α directly bound to HRE1 of the viperin promoter but not to HRE2 under serum starvation or hypoxia (Figure 3D and Supplemental Figure 5E), suggesting that viperin induction was regulated by HIF-1α in the PI3K/AKT/mTOR/HIF-1α signaling pathway.

To investigate whether viperin exerts its metabolic reprogramming function in the TME, we measured the alteration of glucose and fatty acid metabolism under these conditions. The ECAR was significantly reduced in viperin-KD cells compared with MKN28 control cells under serum starvation conditions (Figure 3E), indicating that viperin promoted glucose metabolism of cancer cells in the TME. LD formation was increased in MKN28 control cells but not in viperin-KD cells under serum starvation compared with LD formation under normal conditions (Supplemental Figure 6A). The expression levels of glucose transporters (GLUT1 and -4), major transcriptional regulators (SREBP and ChREBP), and key lipogenic enzymes (ACL, ACC2, and DGAT1) were significantly increased in MKN28 control cells but not in viperin-KD cells under serum starvation compared with expression levels in normal conditions (Figure 3F). The results indicate that lipogenesis in response to serum starvation was driven by viperin expression. To confirm the glucose dependency of viperin-mediated metabolic alteration, we generated MKN28 GLUT4-KD cells, in which GLUT4 expression was suppressed by stable expression of shRNAs (GLUT4 shRNA). The expression levels of GLUT4, SREBP, ChREBP, ACL, and ACC2 were significantly increased in MKN28 control cells but not in GLUT4-KD cells under serum starvation compared with normal conditions (Supplemental Figure 6B), indicating that GLUT-mediated glucose uptake was required for viperin-mediated cancer metabolic reprogramming. Meanwhile, the levels of fatty acid β-oxidation were significantly increased in viperin-KD cells compared with MKN28 controls under serum starvation (Supplemental Figure 6C). In addition, the expression levels of fatty acid transporters (FATP2 and -4, CD36, FABP2 and -4, and CPT1) and a major transcriptional regulator (PPARα) were significantly increased in viperin-KD cells but not in MKN28 control cells under serum starvation compared with expression levels under normal conditions (Supplemental Figure 6D). Similar patterns of metabolic alteration were also observed in MKN28 cells under IFN-γ treatment or hypoxia (Supplemental Figure 6, E and F). The data indicate that viperin enhanced glycolysis and lipogenesis of cancer cells in a glucose-dependent manner in the TME.

### Fatty acids provide crucial negative feedback for viperin induction.

**Figure 4:**
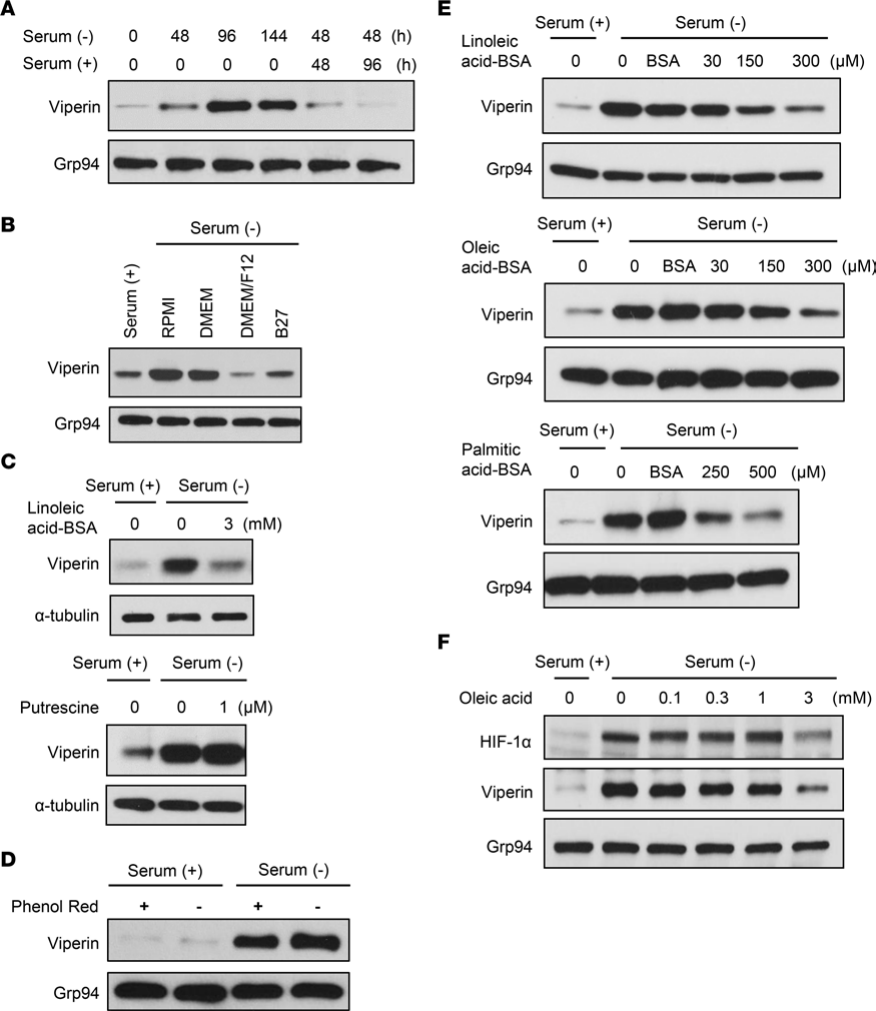
Fatty acid is a negative feedback signal for viperin induction. (**A**) MKN28 cells were cultured in serum-free media and then replenished with serum for the indicated durations. Viperin was detected by immunoblot using MaP.VIP. Grp94 was used as a loading control. (**B**) MKN28 was cultured in the conditioned media, RPMI complete media, serum-free RPMI or DMEM media, DMEM/F12 media, or RPMI supplemented with B27 for 48 hours. Viperin was detected by immunoblotting using MaP.VIP. Grp94 was used as a loading control. (**C**) Linoleic acid, a component of serum, is a key regulator of viperin induction in cancer cells. MKN28 cells were cultured in serum-free media and treated with linoleic acid–BSA or putrescine for 48 hours. Viperin was detected by immunoblotting using MaP.VIP. α-Tubulin was used as a loading control. (**D**) MKN28 cells were cultured in the presence and absence of serum or phenol red for 48 hours. Viperin was detected by immunoblotting using MaP.VIP. Grp94 was used as a loading control. (**E**) Fatty acid is a negative feedback signal for viperin induction. MKN28 cells were cultured in serum-free media and treated with linoleic acid–BSA, oleic acid–BSA, or palmitic acid–BSA for 48 hours. Viperin was detected by immunoblotting using MaP.VIP. Grp94 was used as a loading control. (**F**) MKN28 cells were cultured in serum-free media and treated with oleic acid for 48 hours. Viperin and HIF-1α were detected by immunoblotting using specific mAbs. Grp94 was used as a loading control.

Given that viperin was induced in the TME, we reasoned that the upstream initiators to determine viperin induction could be oxygen and IFNs. To identify the initiators of viperin induction under serum starvation, we monitored the expression levels of viperin in cancer cells with conditioned media. An increase of viperin expression by serum starvation was immediately reversed when the serum was replenished (Figure 4A). The increased level of viperin expression under serum-free RPMI or DMEM conditions was also reversed when DMEM/F12 or B27 supplements were added (Figure 4B). The results indicate that components contained in both DMEM/F12 and B27, but not in RPMI or DMEM, are the upstream initiators that determine viperin induction. To screen the initiator candidates, we analyzed the composition of the media and supplements. Only 2 components, linoleic acid and putrescine, met the criteria. Linoleic acid is an essential polyunsaturated fatty acid for normal growth and development in mammalian cells (44). Putrescine is a precursor for higher polyamine biosynthesis associated with cancer cell growth (45). To identify the component that regulates viperin induction, we incubated cancer cells in serum-free media treated with linoleic acid or putrescine (Figure 4C). The increase in viperin expression by serum starvation was suppressed by treatment with linoleic acid but not putrescine. Since linoleic acid is also included in phenol red in tissue culture media, viperin expression was augmented when cancer cells were incubated in phenol red–free media in the presence and absence of serum (Figure 4D). The results indicate that the deficiency of the serum component linoleic acid initiated viperin induction in the TME. To examine whether viperin suppression by serum replenishment was attributed only to linoleic acid or other common fatty acids, we measured the level of viperin expression in cancer cells in serum-free media treated with linoleic acid, oleic acid, or palmitic acid (Figure 4E). Viperin induction by serum starvation was suppressed in cells treated with each of the 3 fatty acids in a dose-dependent manner. The data indicate that fatty acids provide a negative feedback signal for viperin induction in the TME. Likewise, HIF-1α induction by serum starvation was also suppressed in cells treated with oleic acid (Figure 4F), confirming the mechanism of viperin induction via the HIF-1α signaling pathway under serum starvation.

### Viperin-mediated metabolic reprogramming is required to support CSC properties.

**Figure 5:**
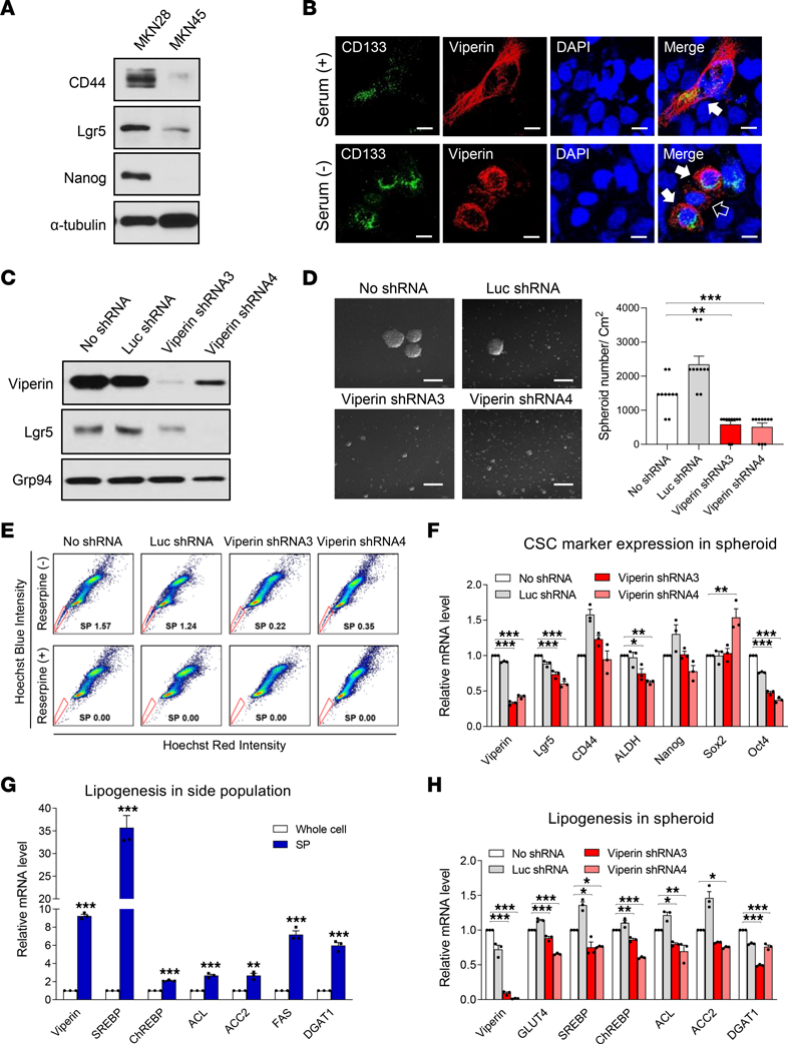
Viperin-mediated metabolic alteration is required to support CSC properties. (**A**) Expression of the CSC markers CD44, Lgr5, and Nanog in MKN28 and MKN45 cells. α-Tubulin was used as a loading control. (**B**) MKN28 cells cultured in the presence or absence of serum were stained with specific mAbs against CD133, a CSC marker, and viperin. Nuclei were stained with DAPI (blue). Solid white arrows indicate viperin expression in CSCs; the open arrow indicates viperin expression in non-CSCs. Scale bars: 10 μm. (**C**) Expression of the CSC marker Lgr5 in MKN28 stable cell lines. (**D**) Single-cell–derived spheroid formation in MKN28 stable cell lines. Shown are representative images of spheroid formation in the stable cell lines. Scale bars: 100 μm. Graph shows quantification of spheroids. Spheroid diameters of greater than 50 μm were counted. Data are presented as the mean ± SEM for the number of spheroids in 10 frames of each sample (*n* = 2). (**E**) Analysis of the SP in MKN28 stable cell lines. Cells were stained with Hoechst 33342 and analyzed using flow cytometry. SPs not stained with Hoechst were gated (red line), and the percentage of SPs is indicated. Reserpine-treated cells were used as negative controls for SPs. (**F**) Expression of CSC markers in spheroids of MKN28 stable cell lines. Relative mRNA levels of viperin and CSC markers (Lgr5, CD44, ALDH, Nanog, Sox2, and Oct4) in spheroids of these cell lines were measured by qRT-PCR and normalized to *ACTB* mRNA. Data are presented as the mean ± SEM (*n* = 2 in triplicate). (**G** and **H**) Comparison of lipogenesis between whole cells and SP cells (**G**) and in spheroids of the stable cell lines (**H**). Relative mRNA levels of the indicated genes in these cell lines were measured by qRT-PCR. Data are presented as the mean ± SEM (*n* = 2 in triplicate). *P < 0.05, **P < 0.01, and ***P < 0.001, by Student’s *t* test (**G**) or 1-way ANOVA with Dunnett’s multiple-comparison test (**D**, **F**, and **H**).

Although viperin expression was increased in the TME, we observed its basal expression in various cancer cell lines including MKN1, MKN28, AGS, HCC4017, and Hs578T under normal conditions (Figure 1D). Cancers are composed of a heterogeneous population of transformed cancer cells. To determine whether a basal expression of viperin could be observed in all types of cancer cells or only in certain types of cancer cells, we monitored cell populations expressing viperin in MKN28 cells by flow cytometry (Supplemental Figure 7A). Interestingly, we observed that viperin was expressed in only a small population (~1%) of MKN28 cells. Likewise, only a small number of viperin-expressing cells was observed in MKN1, MKN28, and AGS cells by immunofluorescence (Supplemental Figure 1D). This suggests that viperin was specifically expressed only in a certain type of cancer cell under normal conditions. We also observed a basal expression of HIF-1α as well as of viperin in MKN28 cells under normal conditions (Figure 3B and Supplemental Figure 5D). It is known that HIF-1α is selectively activated in CSCs and has an essential function in maintaining CSCs under normoxia (46). CSCs are a small population of cancer cells with the capacities of self-renewal, differentiation, and chemoradiotherapy resistance (47, 48). Viperin was detected in HIF-1α–expressing cells of the MKN28 cell line under both normal and serum-starved conditions (Supplemental Figure 7B). Therefore, our data suggest the possibility that the small population of cancer cells expressing viperin in the cancer cell lines under normal conditions might be CSCs. To verify this, we monitored viperin expression along with expression of CSC markers such as CD133, CD44, Lgr5, and ALDH (49) and of the pluripotent transcription factors Nanog, Sox2, and Oct4 (50). The expression levels of CD44, Lgr5, and Nanog were high in MKN28 cells compared with those in MKN45 cells (Figure 5A), suggesting that viperin expression in the cancer cell lines was associated with CSCs. Viperin expression was indeed detected in CD133^+^ cells in the MKN28 cell line under normal conditions (Figure 5B). Moreover, the expression levels of both viperin and CD133 were increased in CD133^+^ cells under serum starvation, and viperin expression was also detected in CD133^–^ cells of the MKN28 line in this condition (Figure 5B). Similarly, the small population of cells expressing both viperin and CD44 (viperin^+^CD44^+^) was observed in MKN28 cells under normal conditions (Supplemental Figure 7C). The populations of viperin^+^CD44^+^ cells and viperin^+^CD44^–^ cells were increased in MKN28 cells under serum starvation (Supplemental Figure 7C). These results indicate that viperin was expressed in CSCs under normal conditions, and its expression is increased in both CSCs and non-CSCs in the TME. To investigate whether viperin expression directly affects the properties of CSCs, we measured the expression levels of CSC markers, single-cell–derived spheroid formation, and the side population (SP) in MKN28 control cells and viperin-KD cells. The spheroid-forming assay allows the evaluation of self-renewal and differentiation at the single-cell level (51), whereas the SP assay allows the isolation of cells that pump Hoechst dye out via ATP-binding cassette transporters (52). The SP exhibits CSC characteristics and has the ability to expel anticancer drugs, thus accounting for the drug resistance in cancer (52). The expression level of the CSC marker Lgr5 was decreased in MKN28 viperin-KD cells compared with that in MKN28 control cells (Figure 5C). Single-cell–derived spheroid formation was also reduced in viperin-KD cells of the MKN28, MKN1, and AGS lines compared with that in their controls (Figure 5D and Supplemental Figure 7D). The size and number of spheroids were decreased in viperin-KD cells. The portion of SP was markedly diminished in viperin-KD cells of the MKN28 and MKN1 lines compared with that in their controls (Figure 5E and Supplemental Figure 7E). These results indicate that viperin expression was essential for the acquisition of CSC properties such as CSC marker expression, self-renewal, and drug efflux. To validate the results from each assay used in this study, we analyzed the correlation between CSC properties. The expression levels of CSC markers in spheroids isolated from viperin-KD cells were reduced compared with those in spheroids from MKN28 controls (Figure 5F), indicating that the self-renewal property of CSCs to form spheroids is proportionally correlated to the expression of CSC markers. HIF-1α expression was increased along with the subsequent viperin expression in single-cell–derived spheroids compared with that in monolayer cells under normal conditions (Supplemental Figure 7F), indicating that HIF-1α expression affects the capacity of CSCs to form spheroids. In addition, spheroid formation derived from single cells of the SP was highly increased compared with that of the non-SP in MKN28 cells (Supplemental Figure 7G), indicating that the SP is indeed CSCs with self-renewal ability. Last, to investigate viperin-mediated metabolic effects on CSCs, we measured the expression levels of metabolic genes in the SP and spheroids isolated from MKN28 cells. The expression levels of viperin, major transcriptional regulators (SREBP, ChREBP), and key lipogenic enzymes (ACL, ACC2, FAS, and DGAT1) were dramatically increased in the SP isolated from MKN28 cells compared with those in MKN28 whole cells under normal conditions (Figure 5G). Moreover, the expression levels of GLUT4, SREBP, and ChREBP and key lipogenic enzymes were significantly reduced in spheroids isolated from viperin-KD cells (Figure 5H). These results suggest that viperin-mediated cancer metabolism is required for the maintenance of CSC properties under normal conditions and enhances its properties in the TME.

### Viperin drives the metabolic phenotype and cancer progression in vivo.

**Figure 6:**
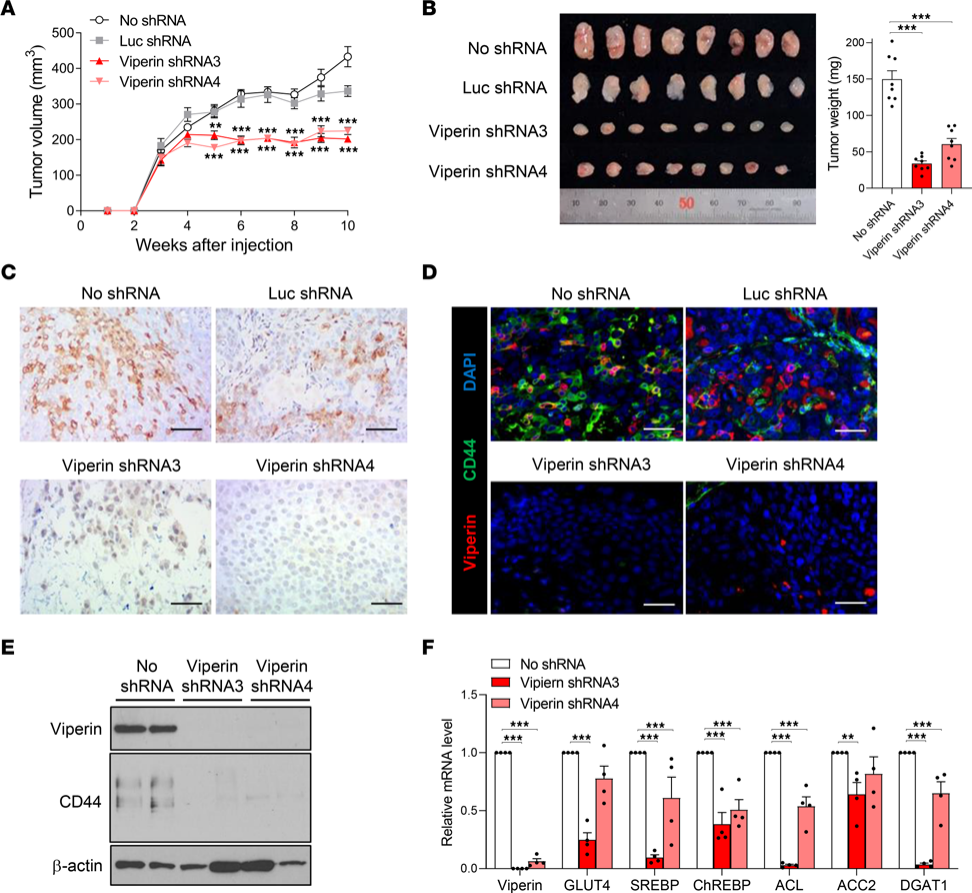
Viperin drives the metabolic phenotype and cancer progression in vivo. (**A** and **B**) Tumor growth in the MKN28 control and viperin-KD cell–derived xenograft mouse models. (**A**) Spheroids of the stable cell lines were dissociated and counted. A single-cell suspension was mixed with an equal volume of Matrigel. The mixture (1 × 10^4^ cells/mouse) was injected subcutaneously into the flanks of 6-week-old male nude mice (*n* = 8/cell line). Tumor growth was monitored weekly, and the tumor volume was measured using a metric caliper. (**B**) After 10 weeks, mice were sacrificed, and tumors were isolated. Tumor size and weight were measured. Data are presented as the mean ± SEM (*n* = 8). (**C** and **D**) IHC staining for viperin (**C**) and immunofluorescence staining for viperin and CD44 (**D**) in tumors isolated from the stable cell–derived xenograft mouse models. Tissue sections were stained with specific mAbs against CD44, a CSC marker, and viperin. Nuclei were stained with DAPI (blue). Scale bars: 100 μm. (**E**) Expression of viperin and CD44 in the isolated tumors. Each protein was detected by immunoblotting using specific mAbs. β-Actin was used as a loading control. (**F**) Lipogenesis in the isolated tumors. Relative mRNA levels of the indicated genes in tumors were measured by qRT-PCR and normalized to *ACTB* mRNA. Data are presented as the mean ± SEM (*n* = 4 in triplicate). **P < 0.01 and ***P < 0.001, by 1-way ANOVA with Dunnett’s multiple-comparison test (**A**, **B**, and **F**).

CSCs are associated with significantly enhanced tumorigenicity. To examine whether viperin-mediated cancer metabolism affects the capacity of tumorigenesis of CSCs in vivo, we monitored tumor growth in mice inoculated with CSCs of MKN28 control and viperin-KD cells (Figure 6 and Supplemental Figure 8). Spheroids or SPs were isolated from these cell lines and dissociated into single cells. The nude mice were injected subcutaneously with the dissociated single cells (1 × 10^4^ cells/mouse), and tumor volume was periodically measured. The mice inoculated with spheroid cells of MKN28 viperin-KD cells exhibited slow tumor growth rates and small tumor volumes compared with MKN28 controls (Figure 6A). The tumors were excised, sized, and weighed 10 weeks after inoculation. Tumor size and weight of mice inoculated with spheroid MKN28 viperin-KD cells were significantly decreased as compared with MKN28 controls (Figure 6B). Similar patterns of tumor growth were observed in mice inoculated with only 1×10^3^ cells from spheroids (Supplemental Figure 8, A and B). In addition, tumor growth of mice inoculated with SP cells (1 × 10^4^ cells/mouse) of MKN28 was elevated compared with mice inoculated with non-SP cells of the MKN28 line or with MKN28 viperin-KD whole cells (Supplemental Figure 8, C and D). These data indicate that viperin expression increased the tumorigenic capacity of CSCs and promoted cancer progression. We also performed IHC on the tumor tissues isolated from mice. Viperin was highly expressed in tumor tissues of MKN28 control cells but not in those of MKN28 viperin-KD cells (Figure 6C), confirming viperin induction in the TME. In addition, immunofluorescence and immunoblot analyses showed that a CSC marker, CD44, was strongly expressed in tumor tissues of MKN28 controls but not in MKN28 viperin-KD cells (Figure 6, D and E), and viperin expression was detected in both CD44^+^ CSCs and CD44^–^ non-CSCs in tumor tissues of MKN28 controls (Figure 6D). Moreover, the expression levels of viperin, GLUT4, SREBP, ChREBP, and key lipogenic enzymes were significantly reduced in tumor tissues of mice with MKN28 viperin-KD cells compared with levels in MKN28 control cells (Figure 6F). These results prove that viperin expression was increased in cancer cells in the TME, enhanced the properties of CSCs, and drove cancer metabolic reprogramming to promote cancer progression.

## Discussion

Metabolic reprogramming is characterized by upregulation or downregulation of metabolic pathways such as glycolysis, lipid metabolism, and glutaminolysis, which provides cancer cells with essential energy and metabolites to facilitate their proliferation and survival in the harsh TME including hypoxia, nutrient deprivation, and cytokine secretion (1, 2, 4). Here, we show that the ISG-encoded protein viperin controls cancer metabolic reprogramming to promote cancer progression. Given that IFNs and most ISGs induce favorable responses to anticancer immunity, which must be overcome for cancer cells in the TME (53, 54), viperin has a distinct function in cancer cells when compared with other ISGs. Although a specific subset of ISGs (including IFI27, ISG15, BST2, OAS1, OAS3, and OASL) that comprise an IFN-related DNA damage–resistant signature (IRDS) are upregulated in cancer cells and induce an unfavorable response to anticancer immunity (55–57), their functions are not associated with cancer metabolism. Our data indicate that viperin is the ISG that controls cancer metabolism.

Viperin is highly expressed in a variety of human cancer tissues compared with expression in normal tissues. Although this protein is expressed in some patients with cancer (20%–40%), its expression level is inversely proportional to their survival rate. Moreover, high expression of viperin results in worse disease-free survival rates in patients with advanced-stage cancer, indicating that viperin expression correlates with cancer progression. Our data suggest that viperin-driven metabolic reprogramming can have negative consequences for most cancer types including gastric, lung, breast, renal, pancreatic, and brain cancers. In melanoma, viperin expression levels are proportional to the survival rate of patients with cancer (58), however its positive function in melanoma remains to be explored.

Interestingly, although we found that viperin was expressed in various human cancer cell lines, it was not expressed in cancer cell lines such as MKN45 or A549, which had high basal lipid levels. However, viperin expression could be induced and drove lipid synthesis in these cell lines when they lacked lipids, such as under serum starvation. In normal conditions, we found that viperin was expressed only in a small population of cancer cell lines such as MKN28 and MKN1 cells, in which basal lipid levels were low. Our study revealed that the small number of cells expressing viperin were in fact CSCs. These data suggest that the basal lipid level required for each cancer cell line differs and that viperin is induced to support demands for lipids in cancer cells.

We also identified initiators and the mechanisms by which viperin was induced in the TME. Cancer cells are under the influence of cytokines including IFN-γ produced by immune cells in the TME and reside in a nutrient- and oxygen-poor environment (34, 35). Treatment with IFN-γ induced viperin expression via the STAT3-mediated IFN signaling pathway. Surprisingly, nutrient deprivation also induced viperin expression via the PI3K/AKT/mTOR/HIF-1α signaling pathway. Interestingly, under serum starvation, external replenishment of fatty acids reduced viperin expression in cancer cell lines. We used supplements such as DMEM/F12 and B27 to screen factors initiating viperin induction during serum starvation and found that linoleic acid deficiency initiated viperin induction, thereby providing evidence that viperin induction is determined by the level of fatty acids, acting as a crucial negative feedback signal in the TME. However, we cannot exclude the possibility that other factors along with fatty acids could also regulate viperin induction in serum starvation conditions, given that serum contains multiple components that are not included in the supplements. Last, oxygen deficiency induced viperin expression via the HIF-1α signaling pathway. Moreover, the ChIP assay showed that HIF-1α directly bound to the viperin promoter and upregulated viperin expression under hypoxia and serum starvation. Given that HIF-1α mediates metabolic alteration in cancers (59, 60), we believe that viperin is a novel downstream molecule of the HIF-1α signaling pathway. The results suggest that viperin induction via the PI3K/AKT/mTOR/HIF-1α pathway as well as the JAK/STAT pathway allowed cancer cells to rapidly adapt to the changing conditions in the TME through metabolic reprogramming.

We show that viperin under IFN-γ treatment, nutrient deprivation, and oxygen-poor conditions enhanced lipogenesis and glycolysis in cancer cells. However, we did not observe viperin-driven metabolic reprogramming in glucose-free media, indicating that viperin regulates cancer metabolism in a glucose-dependent manner. Our finding also suggests that cancer cells conserve energy rather than use it for lipid synthesis when the glucose supply is scarce in the TME. Given that many cancer cells have high rates of glycolysis (61, 62) and compete with immune cells for glucose uptake in the TME (63), viperin’s dependence on glucose to function might be a strategy used by cancer cells to proliferate and survive in harsh conditions. In addition, our analysis of cancer cells expressing viperin mutants provides evidence that the mitochondrial targeting of viperin and the Fe-S cluster–binding motif of viperin are essential for its function to regulate cancer metabolism, suggesting their potential as therapeutic targets in various cancers.

In normal conditions, viperin was expressed in a small number of cancer cells. HIF-1α as an upstream molecule of viperin was expressed at basal levels in cancer cell lines under normoxia. Given that CSCs typically represent a small proportion of total cancer cells and that HIF-1α is selectively activated in CSCs to maintain their properties under normoxia (46), we anticipated that the small number of cancer cells expressing viperin in normal conditions would be CSCs. We show that viperin expression was pivotal for the maintenance of CSCs in normal conditions. Viperin deficiency led to lower expression of CSC markers and reduced the number and size of spheroids as well as the proportion of SPs. Moreover, viperin-deficient spheroids had lower expression of CSC markers, and the sphere-forming capacity of SPs was higher than that of non-SPs. Importantly, viperin deficiency also decreased lipogenesis in CSCs. These data suggest the possibility that viperin-driven metabolic changes are required for the maintenance of CSC properties in normal conditions. Moreover, under serum starvation, viperin expression was increased in CSCs and induced in neighboring cancer cells. Taken together, our data suggest that an increase of viperin expression in the TME upregulated metabolic reprogramming of CSCs as well as non-CSCs, which in turn activated CSC properties and enhanced the proliferation of cancer cells, resulting in their rapid adaption to and survival in changing conditions. Finally, we show that viperin expression in cancer cells, including CSCs, plays a major role in cancer progression. Only 1,000 cancer cells from spheroids expressing viperin, which were inoculated into nude mice, were required to lead to cancer formation. An increase in the number of cells inoculated from spheroids or SPs expressing viperin enhanced the growth rate of cancer. Conversely, cancer cells from viperin-deficient spheroids or non-SPs were defective for cancer formation and growth. In addition, cancer tissues lacking viperin expression exhibited reduced numbers of CSCs and lower levels of lipogenesis. The data demonstrate that viperin-driven metabolic reprogramming activated CSCs and neighboring cancer cells to promote cancer formation and progression (Supplemental Figure 9). Therefore, the IFN-inducible protein viperin is not only an attractive target for cancer metabolism–based therapeutics but also a potential target for combating drug-resistant CSCs in anticancer therapies.

In conclusion, we show that viperin was highly expressed in cancer tissues from patients with gastric, lung, or breast cancer, and primarily in CSCs of several types of cancer. Its expression was upregulated by the deficiency of fatty acids and oxygen as well as by treatment with IFN-γ via the JAK/STAT and PI3K/AKT/mTOR/HIF-1α pathways, respectively. Viperin induction increased lipogenesis and glycolysis via inhibition of fatty acid β-oxidation in cancer cells. An increase of viperin in CSCs promoted metabolic reprogramming and enhanced CSC properties, which facilitated cancer progression in vivo. Our data indicate that the ISG-encoded protein viperin drove metabolic alteration to support cancer proliferation, growth, and survival, with potentially important implications for the development of anticancer therapeutics targeting cancer metabolism as well as IFN responses.

## Methods

The methods are described in detail in Supplemental Methods.

### Human tissue samples.

Tissue microarray (TMA) slides were purchased from SuperBioChips. TMAs for stomach (CQ2 and CQN2) included 58 tumor cores (*n* = 45 adenocarcinoma samples, *n* = 8 signet ring cell carcinoma samples, and *n* = 5 samples of other types of stomach cancer) and 56 normal tissue cores. TMA for lung (CC5, CCN5, and CCA4) included 97 tumor cores (*n* = 15 adenocarcinoma samples, *n* = 49 squamous cell carcinoma samples, and *n* = 33 samples for other types of lung cancers) and 68 normal tissue cores. TMAs for breast (CBA4 and CBB3) included 55 tumor cores (*n* = 51 invasive ductal carcinoma samples and *n* = 4 samples of other types of breast cancers) and 23 normal tissue cores. TMAs for gastric cancer also included 121 samples of advanced gastric cancer (pT ≥3) from patients who underwent gastrectomy at the Asan Medical Center (Seoul, Korea). One 2 mm diameter tissue core was obtained from each formalin-fixed, paraffin-embedded tumor block.

### IHC staining and scoring of TMA.

IHC was performed on TMA slides as described elsewhere. The TMA block was cut into 5 μm sections and deparaffinized in xylene, followed by rehydration through a graded alcohol series. Antigen retrieval was performed using a pressure chamber (DAKO) with pH 6.0 Target Retrieval Solution (DAKO, S2369). Endogenous peroxidase activity was blocked by incubation in 3% H_2_O_2_ for 10 minutes. The sections were incubated with a specific mAb against viperin (MaP.VIP) for 1 hour at room temperature. Subsequently, the antigen-Ab reaction was highlighted with the EnVision+ Dual Link System-HRP and DAB+ (DAKO, K4065). TMA sections were lightly counterstained in a Mayer’s hematoxylin bath (DAKO, S3309) and then examined by light microscopy. A negative control IgG was used in place of a primary Ab to evaluate nonspecific staining, and the TMA included appropriate positive control specimens. Two dedicated gastrointestinal pathologists evaluated the IHC data independently and were blinded to the clinical data. Viperin expression levels were assessed using a combinative semiquantitative scoring method. All TMA cores were classified into the following 4 score groups according to viperin expression levels: 0 (≤5% weak staining); 1+ (6%–50% weak staining or ≤5% moderate/marked staining); 2+ (51%–100% weak staining or 6%–50% moderate/marked staining); and 3+ (>51% moderate/marked staining) (Supplemental Figure 1A). The median value was used as a cutoff between the high and low expression groups. Samples with a score above 1 (median value) were considered to have high expression, and samples with score of 1 or less were considered to have low expression.

### Cancer cell lines, Abs, and reagents.

Gastric cancer cell lines (MKN1, MKN28, MKN45, AGS, SNU668, SNU601, Hs746T, SNU484, NCC19, and NCC20) were provided by Jae-Ho Cheong (Yonsei University, Seoul, Korea) and Hyunki Kim (Yonsei University, Seoul, Korea). Lung cancer cell lines (A549, SK-LU-1, HCC4017, and HCC2279) were provided by Yun-Han Lee (Keimyung University, Daegu, Korea) and Hyun Seok Kim (Yonsei University, Seoul, Korea). Breast cancer cell lines (MCF-7, BT-474, Hs578T, and SK-Br-3) were provided by Sang-Kyu Ye (Seoul National University, Seoul, Korea) and Kyung-Hee Chun (Yonsei University, Seoul, Korea). The identity of the cancer cell lines (MKN28 and MKN45) was confirmed by DNA sequencing performed by the Korea Cell Line Bank (Seoul, Korea). All cancer cell lines used in this study were cultured in RPMI-1640 (Hyclone, SH30027.01) or DMEM (Hyclone, SH30243.01) supplemented with 10% FBS (Hyclone, SH30919.03) and 1% penicillin/streptomycin (Hyclone, SV30010) at 37°C in an incubator containing 5% CO_2_. For hypoxia experiments, cells were placed in a hypoxic chamber containing 1% O_2_, 5% CO_2_, and 94 % N_2_.

The mouse mAb against viperin (aa 26–277) (MaP.VIP) was described previously (23, 24). The following Abs were used: rabbit mAbs against TOMM20 (Abcam, ab186735); STAT3 (Cell Signaling Technology, 30835s); p-STAT3 (Y705) (Cell Signaling Technology, 9145s); PTEN (Cell Signaling Technology 9559s); AKT (Cell Signaling Technology, 4691s); p-AKT (S473) (Cell Signaling Technology, 9271s); S6 (Cell Signaling Technology, 2217s); p-S6 (S235/236) (Cell Signaling Technology, 4858s); HIF-1α (Cell Signaling Technology, 14179s); CD44 (Cell Signaling Technology, 3570s); and Nanog (Cell Signaling Technology, 4903s). The mouse mAbs against Lgr5 (Abcam ab75850); β-actin (MilliporeSigma A5316); and α-tubulin (MilliporeSigma T6199) were also used. The rat mAb against Grp 94 (ADI-SPA-850-F) was obtained from Enzo Life Sciences. Goat anti–mouse Ig (115-035-146); anti–rabbit Ig (111-035-144); and anti–rat Ig (112-035-167) secondary Abs were purchased from Jackson ImmunoResearch.

IFN-γ (Thermo Fisher Scientific, PHC4031) and LY294002 (Merck Millipore, 440202) were used. Ranolazine dihydrochloride (R6152); cobalt chloride (CoCl_2_) (15862-1ML-F), 2-ME (M6383); rapamycin (R0395); S31-201 (SML0330); SF1670 (SML0684); oleic acid (O3008); linoleic acid (L9530); palmitic acid (A7922); putrescine (P7505); and reserpine (R0875) were purchased from MilliporeSigma. The 2.4G2 blocking buffer was provided by Chae Gyu Park (Yonsei University, Seoul, Korea).

### In vivo xenograft experiments.

Five-week-old male BALB/c nude mice were purchased from Central Lab Animal Inc. Dissociated spheroids and SP cells were counted, resuspended in 50 μL PBS (Hyclone, SH30028.02), and mixed with an equal volume of Matrigel (Corning 354248). The mixture was injected subcutaneously into the flanks of 6-week-old male nude mice. Tumor growth was monitored weekly and measured using a metric caliper. Tumor volume was calculated as 1/2 × L × W^2^, where L stands for the length, and W for the width measured by a caliper in millimeters. After 8–10 weeks, mice were sacrificed, and tumors were isolated, measured, and weighed. The isolated tumors were resected, fixed in 10% neutral buffered formalin, and embedded in paraffin for sectioning on a rotary microtome, followed by slide mounting and histologic assessment.

### IHC analysis of xenograft tumors.

Samples were deparaffinized and rehydrated through gradually descending series (100%, 95%, and 70%) of ethanol. Antigen retrieval (DAKO) was performed using a pressure cooker. After cooling on ice for 1 hour, the sections were incubated in 3% H_2_O_2_ for 30 minutes to block endogenous peroxidase activity. The sections were washed twice with PBS and incubated with Protein Block Serum Free (DAKO, X0909) for 1–2 hours at room temperature to reduce nonspecific signals. The Mouse on Mouse Kit (Vector Laboratories, BMK-2202) was then used according to the manufacturer’s instructions. The sections were incubated with primary Abs overnight at 4°C. After washing 3 times with PBS, sections were incubated with HRP-conjugated secondary Abs (DAKO P0447) for 15 minutes at room temperature. DAB (DAKO) was used for the development of Abs, and Mayer’s Hematoxylin (DAKO) was used for counterstaining. Each experiment was performed with identical durations for DAB development.

### Statistics.

The data are presented as the mean ± SEM. Statistical significance was determined using an unpaired, 2-tailed Student’s *t* test. Comparisons of more than 2 groups were calculated using 1-way ANOVA with Dunnett’s multiple-comparison test or Tukey’s multiple-comparison test. A *P* value of less than 0.05 was considered statistically significant. For the TMA data, statistical analyses were performed using PASW Statistics 18 for Windows (IBM SPSS, version 18.0). Kaplan-Meier and Cox regression tests were applied to analyze the survival data. Statistical significance was defined at a *P* value of less than 0.05. Analysis of published data sets for gene expression and survival rates was performed using cBioPortal for Cancer Genomics (http://www.cbioportal.org). In brief, The Cancer Genome Atlas (TCGA) data sets were selected, and a query for viperin was performed. An mRNA expression *z* score ± 1.5 was set as the threshold. Samples with a *z* score above 1.5 were considered to have high expression, and samples with a *z* score below –1.5 were considered to have low expression. The other samples were considered to have no alteration. The survival rates of patients with cancer were analyzed by Kaplan-Meier method, and the statistical significance of survival time was determined by the log-rank test. A *P* value of less than 0.05 was considered statistically significant. The data sets used in this study were from 288 patients with gastric adenocarcinoma (26), 230 patients with lung adenocarcinoma (27), 1,981 patients with breast cancer (28), 443 patients with renal cell clear carcinoma (29), 184 patients with pancreatic adenocarcinoma (30), and 206 patients with glioblastoma (31).

### Study approval.

All procedures were conducted in accordance with the guidelines of the Declaration of Helsinki. This study was approved by the IRB of Asan Medical Center, and all patients provided written informed consent. All animal experiments were conducted in accordance with the guidelines of and approved by the IACUC of the Yonsei University Health System.

## Author contributions

JYS conceived and designed experiments. KMC, JJK, JY, KSK, YG, JE, and HJ performed experiments and analyzed data. KK, YSP, and JYC performed TMA experiments and analyzed data. KTN assisted with the design and analysis of in vivo experiments. KMC, JJK, and JYS wrote the manuscript. All authors contributed to the final version of the manuscript.

## Supplementary Material

Supplemental data

## Version 1 10/13/2022

In-Press Preview

## Version 2 12/15/2022

Electronic publication
