# Supplementary material for: The interferon-inducible protein viperin controls cancer metabolic reprogramming to enhance cancer progression
Source: J Clin Invest. 2022 Dec 15;132(24):e157302. doi: 10.1172/JCI157302 (PMC9753993; doi:10.1172/JCI157302)
Supplement: Supplemental data [file jci-132-157302-s018.pdf]

Supplemental Methods

Immunoblot analysis. Cell pellets were lysed in 1% Triton X-100 in TBS (AMRESCO 0694) supplemented with protease inhibitor cocktail (cOmplete Mini, F. Hoffmann-La Roche 11697498001) and phosphatase inhibitors (PhosSTOP, F. Hoffmann-La Roche 04906845001). Protein concentration was measured by a bicinchoninic acid assay (Thermo Fisher Scientific 23227). The proteins were separated by 12% SDS-PAGE and transferred to PVDF membranes (Merck Millipore IPVH00010). The blots were blocked in 5% non-fat milk (MP Biomedicals 0290288705) and 0.05% Tween (VWR 97063-872) in PBS for 1 h, incubated with primary antibodies for 16 h at 4℃, probed with anti-Ig horseradish-conjugated secondary antibodies for 1 h at room temperature, and incubated with enhanced chemiluminescence reagents (Thermo Fisher Scientific 34080s). Grp94, α-tubulin, or β-actin was used as a loading control.

Immunofluorescence. Cells were grown in 24-well tissue culture plates (SPL 30024) containing a 13-mm-diameter coverslip (SUPERIOR 111520). The coverslips were fixed with 3% paraformaldehyde (Sigma-Aldrich P6148) in PBS for 45 min at room temperature. After fixation, the cells were washed in PBS and permeabilized with 0.1% Triton X-100 and 0.01% SDS (GenDEPOT S0793-100) in PBS for 5 min at room temperature. The cells on coverslip were blocked with 0.2% Tween in PBS containing 10% normal goat serum (Thermo Fisher Scientific 16210064) for 20 min at room temperature and incubated with primary antibody for 1 h at room temperature. Following washing with 0.2% Tween in PBS, the cells were incubated with goat anti-rabbit or anti-mouse Ig secondary Ab diluted in 0.2% Tween in PBS containing 2.5% normal goat serum for 30 min at 37℃. After washing with 0.2% tween in PBS, the coverslips were rinsed once in PBS and mounted with ProLong Gold Antifade reagent (Invitrogen P36930). For lipid droplet (LD) staining, the fixed cells were permeabilized with 0.5% saponin (Sigma-Aldrich 47036) in

PBS for 10 min. Following washing with 0.2% Tween in PBS, the cells were incubated with bodipy-FITC (Molecular Probes D3922) in 150 mM NaCl (Samchun Chemicals S0484) for 20 min at room temperature. After washing with 0.2% tween in PBS, the coverslips were rinsed once in PBS and mounted with ProLong Gold Antifade reagent. Images were acquired with a Carl Zeiss LSM700 confocal microscope.

For immunofluorescence staining of TMA, representative images were captured using EVOS M5000 (Invitrogen) for each specimen. The overall score of the staining intensity had four categories: negative (0), mild (1+), moderate (2+), and marked (3+) expression. Co-localization between viperin and TOMM20 was analyzed in all tissue specimens, and samples were classified into the positive and negative tissues according to the presence or absence of co-localization.

shRNA knockdown of viperin and GLUT4. pLKO.1-Puro lentiviral plasmids (Sigma-Aldrich SHC004) with short hairpin sequences targeting viperin were generated as described previously (24). Several candidate target sequences were obtained from Dharmacon: ORF1 (5′-GAGAATACCTGGGCAAGTT-3′), ORF2 (5′-TAGAGTCGCTTTC AAGATA-3′), ORF3 (5′-GGAGTAAGGCTGATCTGAA-3′), UTR1 (5′-GAAATAAGCTC TAGTGATA-3′), and UTR3 (5′-CGAATAACTTGGATAGCAA-3′). To increase knockdown efficiency, two lentiviral plasmids including different target sequences were combined: Viperin shRNA1 (ORF2), Viperin shRNA2 (ORF1 and ORF3), Viperin shRNA3 (ORF3 and UTR3), and Viperin shRNA4 (ORF3 and UTR1). As a negative control, a target sequence to luciferase Luc shRNA (5′-CGTACGCGGAATACTTCGA-3′) was used. pGIPZ lentiviral plasmids with the short hairpin target sequences to GLUT4 were used (Open Biosystems TLP4614) (23). To increase knockdown efficiency, two lentiviral plasmids including different target sequences were combined: GLUT4 shRNA (clone ID: V3LHS_376728 and V3LHS_376730). For packaging and envelopment, the

pΔVPR plasmid and the pVSVG plasmid were also used. HEK-293T cells were co-transfected with three plasmids, 12 μg of the lentiviral plasmid, 12 μg of the pΔVPR plasmid and 12 μg of the pVSVG plasmid, by using lipofectamine 2000 (Thermo Fisher Scientific 11668-019), and incubated at 37℃ for 16-24 h. The co-transfected cells were shifted to 32℃. After 1 day, supernatants containing lentiviruses were collected every 16-24 h three times. Cancer cell lines (MKN1, MKN28, and AGS) were spin infected with the supernatants containing viruses for 90 min (1,455 × *g*) at 32℃ in the presence of 8 μg/ml polybrene (Merck Millipore TR-1003-G) three times and incubated at 32℃. After 1 day, the infected cells were shifted to 37℃ and selected with puromycin. The knockdown efficiency was assessed by western blot or RT-PCR analysis.

siRNA knockdown of HIF-1α. siRNAs targeting HIF-1α were purchased from Bionics (Seoul, Korea). The sequences for siRNAs were as follows: HIF-1α siRNA1 (5′-GATGGAAGCACTAGACAAA-3′), HIF-1α siRNA2 (5′-GGACACAGATTTAGACTTG-3′), and scrambled siRNA as a negative control (5′-TTCTCCGAACGTGTCACGT-3′). Cells were transfected with each siRNA construct by using oligofectamine (Thermo Fisher Scientific, 12252011), and incubated at 37℃ for 24 h. The transfected cells were cultured in the presence and absence of serum for 48 h or hypoxia chamber for 24 h. The knockdown efficiency was assessed by immunoblot analysis.

Generation of cancer cell lines stably expressing viperin. pBMN-IRES-Neo retroviral plasmid was provided by Dr. Peter Cresswell (Yale University). The wild type viperin or mutant viperin (DCA, substitution of cysteine residues 88 and 91 to alanine residues) was cloned into pBMN-IRES-Neo. Chimeric viperin constructs were also generated in which residues 1–42 of wild type viperin and mutant viperin (DCA) were replaced by the residues 1–34 (mitochondrial localization sequence, MLS) of vMIA (23, 24) and then cloned into pBMN-IRES-Neo to yield

Viperin, Viperin (DCA), MLS-Viperin, and MLS-Viperin (DCA), respectively. For packaging, the pCL-Ampho plasmid provided by Dr. Peter Cresswell (Yale University, New Haven) was also used. HEK-293T cells were co-transfected with two plasmids, 12 μg of the retroviral plasmid and 12 μg of the pCL-Ampho plasmid by using lipofectamine 2000 and incubated at 37℃ for 16-24 h. The co-transfected cells were shifted to 32℃. After 1 day, supernatants containing retroviruses were collected every 16-24 h three times. Cancer cell line (MKN45) was spin infected with the supernatants containing viruses for 90 min (1,455 × *g*) at 32℃ in the presence of polybrene (8 μg/ml) three times and incubated at 32ºC. After 1 day, the infected cells were shifted to 37ºC and selected with neomycin. The efficiency of stable expression was assessed by western blot or RT-PCR analysis.

RNA extraction, cDNA preparation, and qRT-PCR. Total RNA was extracted from cancer cell lines or tumor tissues by the RNeasy Mini Kit (Qiagen 74106). cDNA synthesis was performed with 1 μg RNA using Prime Script First-Strand cDNA Synthesis Kit (Takara Bio RR036). The cDNA was quantified by real-time quantitative reverse transcription PCR (qRT-PCR) using SYBR Green (Applied Biosystems 4364344). The reaction was performed at 95 ℃ for 10 min, which was followed by a three-step PCR program of 95 ℃ for 30 s, 55 ℃ for 1 min, and 72℃ for 30 s repeated for 50 cycles. The primers used in the qRT-PCR are listed in (Supplemental Table 1). The qRT-PCR was performed in triplicate for each sample. Quantitation was performed by the comparative Ct ($2^{-\Delta\Delta Ct}$) method. The Ct value for each sample was normalized by the value for β-actin gene. Two or three independent experiments were analyzed statistically for differences in the mean values, and the *P* values are indicated in the figures.

Cell proliferation assay. Cells were plated at 1×10^4 cells per well in 6-well plates (SPL 30006) and cultured at 37℃ incubator containing 5% CO_2 for 7 days. At the indicated times, the

number of cells in triplicate wells were determined using the trypan blue exclusion method (Thermo Fisher Scientific 15250-061).

Fatty acid oxidation assay. Fatty acid β-oxidation was measured with fatty acid oxidation complete assay kit (Abcam ab217602) according to the manufacturer's instruction. Briefly, cells were seeded into a black, clear-bottom cell culture plate (SPL 30296) at 5×10^4 cells per well and incubated overnight in 5% CO_2 at 37 °C. The complete media were replaced with glucose deprivation media for 1 day or serum-free media for 2 days. After washing twice with pre-warmed fatty acid-free media, 90 μL fatty acid measurement media and 10 μL extracellular O_2 consumption reagent (Abcam ab197243) were added to each well. Fatty acid measurement medium without cells was used as a signal control. Each well was sealed with 100 μL pre-warmed high sensitivity mineral oil. Signals were read immediately at 1.5 min intervals for 60 min using excitation/emission wavelengths of 380 nm/650 nm in EnVision Multilable Plate Reader (PerkinElmer). The plate was maintained at 37 °C throughout the course of the experiment. The data sets were analyzed statistically for differences in the mean values, and P values are indicated in the figures.

The extracellular acidification rate analysis. The extracellular acidification rate (ECAR) of cancer cell lines was determined using a Seahorse XF96 extracellular flux analyzer (Seahorse Bioscience). Cells were plated at 1×10^4 cells per well and cultured in complete media or serum-free media for 48 h. Prior to measurement, cells were incubated in unbuffered DMEM assay medium (Agilent 103334-100) in a non-CO_2 incubator at 37 °C for 1 h. The DMEM assay medium for ECAR measurement contained 2 mM glutamine (Sigma-Aldrich G7513). The following compounds (Agilent 103020-100) were injected: glucose (10 mM), oligomycin A (1.5 μM), and

2-DG (100 mM). This allowed for calculation of glycolysis rate, glycolytic capacity, and glycolytic reserve. Basal ECAR was measured prior to addition of glucose.

Mitochondrial respiration analysis. The cellular oxygen consumption rate (OCR) of cancer cell lines was determined using a Seahorse XF96 extracellular flux analyzer (Seahorse Bioscience). Cells were plated at 1×10^4 cells per well and cultured in complete media for 48 h. Prior to measurement, cells were incubated in unbuffered DMEM assay medium (Agilent 103334-100) in a non-CO_2 incubator at 37 °C for 1 h. The inhibitors of mitochondrial respiratory chain complexes (Agilent 103015-100) were injected: oligomycin A (1 µM), carbonyl cyanide-4-(trifluoromethoxy)phenylhydrazone (FCCP) (1 µM), and rotenone/antimycin A (0.5 µM). The mitochondrial parameters including basal respiration, ATP production, proton leak, and maximal respiration were calculated from the OCR before and after the addition of inhibitors. Basal OCR was measured prior to addition of oligomycin.

ATP assay. Cellular ATP levels were measured with the ATP bioluminescence assay kit HS II (Roche 11699709001) following the manufacturer's instructions. For each assay 1×10^4 cells/sample were used. The samples were incubated with cell lysis reagent for 5 min at room temperature in darkness and luciferase reagent was added to the samples. The level of cellular *ATP was measured as* the amount of light emission that is *produced* when *luciferase* catalyzes *ATP-dependent* D-luciferin oxidation to oxyluciferin by using Centro XS^3 LB 960 microplate luminometer (EG&G Berthold). The amount of *light emission* expressed as relative light unit (RLU) was converted into the ATP concentration using ATP standard solutions. The data sets were analyzed statistically for differences in the mean values, and *P* values are indicated in the figures.

Chromatin immunoprecipitation assay. For chromatin immunoprecipitation (ChIP) assay, cells were cross-linked with 1% formaldehyde (Sigma-Aldrich F8775) and quenched with 2 M

cold glycine (Bio-Rad Laboratories 161-0724). The cells were then pelleted and lysed in lysis buffer (5 mM PIPES (pH 8.0), 85 mM KCl, and 0.5% NP-40). Nuclei were pelleted and subjected to sonication in Buffer A (10 mM Tris-HCl (pH 8.0), 2 mM EDTA, and 0.2% SDS) using a Bioruptor (Diagenode). Chromatin samples were diluted in Buffer B (10 mM Tris-HCl (pH 8.0), 2% Triton X-100, 280 mM NaCl, and 0.2% deoxycholate). Immunoprecipitations were performed with primary antibody overnight at 4℃. Chromatin-antibody complexes were pulled down with Protein A/G Dynabeads (Thermo Fisher Scientific 10001D/10003D) and eluted with elution buffer (1% SDS and 100 mM $NaHCO_3$). To remove the protein and reversal of crosslinks, Proteinase K (Sigma-Aldrich P6556) were added. The amounts of immunoprecipitated DNA were assessed by real-time PCR. The primers used in this study are listed in (Supplemental Table 2). The primers for viperin (HRE1) and viperin (HRE2) were used to amplify a 200 - 300 base pair fragment corresponding to the viperin promoter. FOXM1 and Untr12 were used as positive and negative controls, respectively.

Flow cytometry. Cells were washed with FACS buffer (1% FBS in PBS) and incubated with 2.4G2 antibody to block Fc receptor on the cell surface for 15 min at 4℃. Then, cells were incubated with APC/Cy7-conjugated anti-CD44 antibody (Biolegend 103027) or isotype control antibody (Biolegend 400623) for 30 min at 4℃ and washed three times with FACS buffer. After washing, cells were fixed in Cytofix fixation buffer (BD biosciences 554655) for 20 min at 4℃. For intracellular staining, cells were permeabilized with Perm/Wash buffer (0.1% saponin in FACS buffer) and incubated with Alexa Fluor 647-conjugated MaP.VIP antibody (Bioss Antibodies BS-8718R-A647) for 30 min at 4℃. After washing three times with FACS buffer, single cell suspensions were obtained with cell strainers and resuspended in FACS buffer. Then

cells were run on a BD LSR-Fortessa X-20 Flow Cytometer (Becton Dickinson). Data were collected and analyzed using FlowJo software.

Side population assay. Side population (SP) assay was performed as described previously with minor modifications (52). Briefly, 1×10^6 cells were resuspended in 1 ml HBSS media (Gibco 14170-112) with 10 mM HEPES (Thermo Fisher Scientific 15630-080) and 2% FBS, and incubated with 5 μg/ml of Hoechst 33342 (Sigma-Aldrich B2261) by rotating at 37°C for 60 min. As a negative control, 50 μM reserpine (Sigma-Aldrich R0875) was added. After incubation, the cells were cooled on ice for 5 min and centrifuged at 100 x *g* for 8 min at 4°C. Hoechst-stained cells were resuspended in ice-cold HBSS media with 10 mM HEPES and 2% FBS, and added with 2 μg/ml of propidium iodide (PI) (Sigma-Aldrich P4170) to exclude dead cells. Side population cells were analyzed and sorted using a BD LSR-Fortessa X-20 Flow Cytometer (Becton Dickinson) and a BD FACSAria II Cell Sorter (Beckman Coulter), respectively. The Hoechst dye was excited at 350 nm and its fluorescence was measured at two wavelengths using a 450/20 nm band pass filter and a 675 nm long pass edge filter. A 610 nm short pass dichroic mirror was used to separate the emission wavelengths. Hoechst "Blue" represents the 450/20 nm filter, the standard analysis wavelength for Hoechst 33342 DNA content analysis. The far right of the Hoechst "Red" (the 675 nm filter) axis indicates cells positive for PI. A live gate of side population was determined on the flow cytometer using Hoechst blue and red axes to exclude dead cells, red cells (no Hoechst stain), and debris. Side population cells were also sorted and reanalyzed to establish high purity (> 98%). Data were collected and analyzed using FlowJo software.

Spheroid formation assay. Cells were plated as single cell suspension at 1×10^4 cells per well in 6-well ultra-low attachment plates (Corning 3471) and cultured in serum-free DMEM/F12 medium (Gibco 11330-032) supplemented with B27 (Gibco 17504044), 20 ng/ml

FGFb (invitrogen PHG0021), 20 ng/ml EGF (invitrogen PHG0311), and 1% penicillin/streptomycin. After two weeks, wells were analyzed for spheroid formation and were quantified using an inverted microscope. Spheroids were collected by centrifugation at 300 × *g* for 5 min and dissociated to singe cell suspension. The single cell suspension was plated at 1×10^4 cells per well in 6-well ultra-low attachment plates and cultured in serum-free conditioned media described above. After two weeks, wells were analyzed for subspheroid formation. Spheroids with a diameter more than 50 μm were counted for the spheroid-forming index.

Supplemental Figure 1

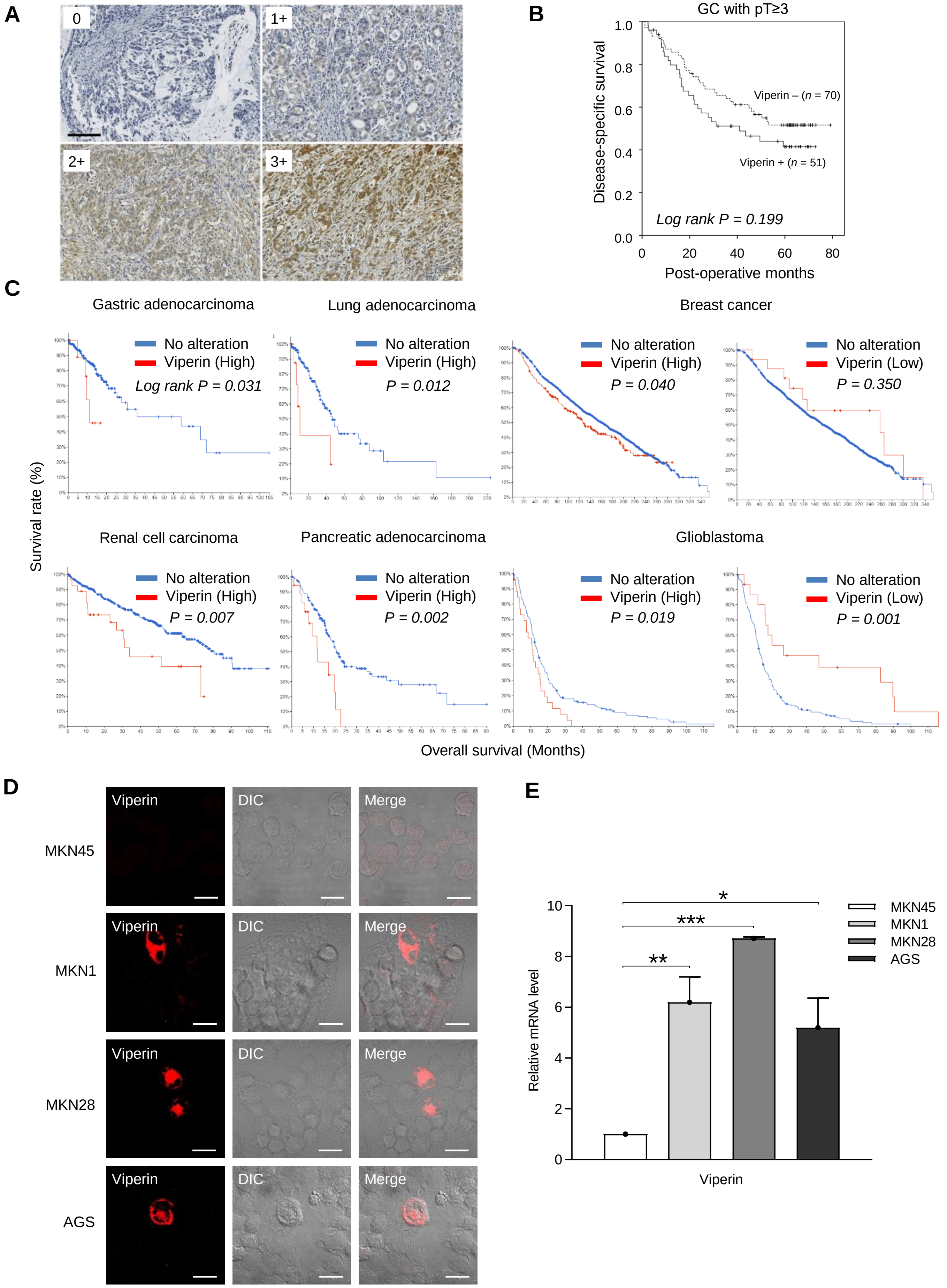

Supplemental Figure 1. The expression level of viperin is associated with the survival rate of patients with cancer. (**A**) Immunohistochemical staining and scores for gastric cancer samples labeled with a specific monoclonal antibody to viperin, MaP.VIP. No expression (score 0), mild expression (score 1+), moderate expression (score 2+), and marked expression (score 3+). Scale bar, 100 μm. (**B**) Disease-specific survival of gastric cancer patients was analyzed in Kaplan-Meier plots. In patients with combined advanced stage cancer (n = 121, pT ≥ 3), patients with high expression of viperin showed a worse disease-free survival trend compared to patients with low expression of viperin. (**C**) The datasets published in cBioPortal for Cancer Genomics were analyzed for the correlation between viperin expression and survival rate of patients with cancer. Kaplan-Meier survival analysis of patients with cancer stratified by viperin expression. Gastric adenocarcinoma (n = 288); lung adenocarcinoma (n = 230); breast cancer (n = 1981); renal cell carcinoma (n = 443); pancreatic adenocarcinoma (n = 184); glioblastoma (n = 206). P-value, log rank test for patients with high or low expression of viperin versus patients with no alteration of viperin expression. (**D** and **E**) Viperin expression in cancer cell lines. (**D**) Immunofluorescence staining of gastric cancer cell lines, MKN45, MKN1, MKN28, and AGS. Viperin (red). Scale bar, 20 μm. (**E**) Relative mRNA levels of viperin in gastric cancer cell lines. The mRNA levels were measured by qRT-PCR and normalized to β-actin mRNA. Data are presented as means ± SEM (n = 3 in triplicate). Statistical analysis was performed by one-way ANOVA with Dunnett's multiple-comparison test (**E**). *$P < 0.05$; **$P < 0.01$; ***$P < 0.001$.

Supplemental Figure 2

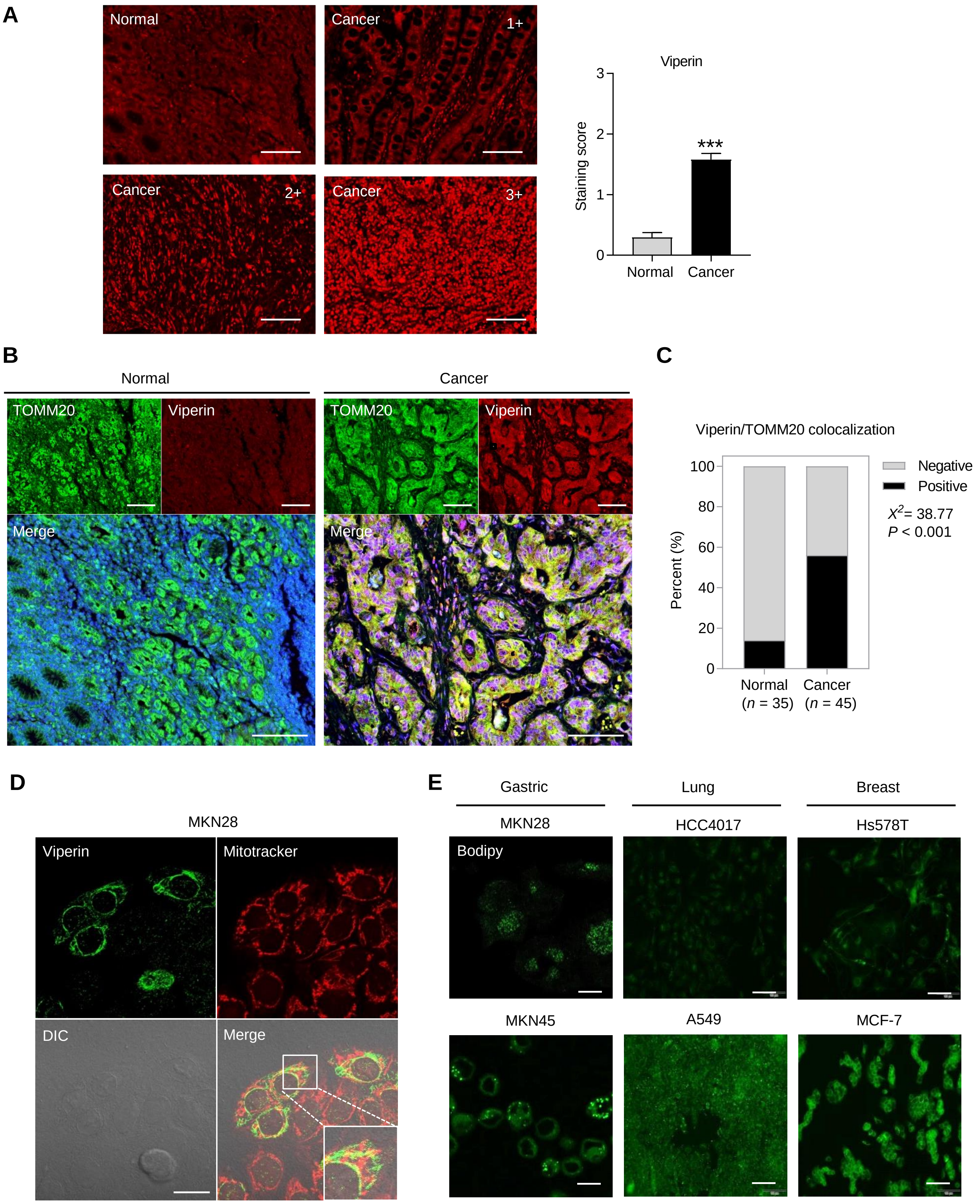

Supplemental Figure 2. Mitochondrial localization of viperin in cancer. (**A**-**C**) Immunofluorescence staining for normal (n = 45) and gastric cancer tissues (n = 35). The paired adjacent non-cancer tissues were used as normal. (**A**) Tissues were stained with monoclonal antibody to viperin (MaP.VIP). Mild expression (score 1+), moderate expression (score 2+), and marked expression (score 3+). Scale bar, 100 μm. Statistical analysis of viperin expression in normal and cancer tissues (t test). Data are presented as means ± SEM. ***$P < 0.001$. (**B**) Tissues were stained with specific monoclonal antibodies to TOMM20, a marker for mitochondria (green) and viperin (red). Scale bar, 100 μm. (**C**) Statistical analysis of viperin localized to mitochondria in normal and cancer tissues. Pearson's χ^2 test and P value for cancer versus normal tissues. Note that viperin was markedly localized to the mitochondria in cancer tissue. (**D**) Subcellular localization of viperin in MKN28. Cells were stained with a specific monoclonal antibody to viperin (green) and MitoTracker Red (red), an indicator for mitochondria. A representative image from individual experiments is shown. Scale bar, 20 μm. Note that viperin was localized to the mitochondria as well as endoplasmic reticulum in cancer cells. (**E**) The basal level of lipid in gastric cancer cell lines (MKN28 and MKN45), lung cancer cell lines (HCC4017 and A549), and breast cancer cell lines (Hs578T and MCF-7). Cells were stained with bodipy-FITC (green), an indicator for lipid droplets (LDs), Scale bar, 100 μm. Note that MKN28, HCC4017, and Hs578T where the basal level of lipid was low, and MKN45, A549, and MCF-7 where the basal level of lipid was high.

Supplemental Figure 3

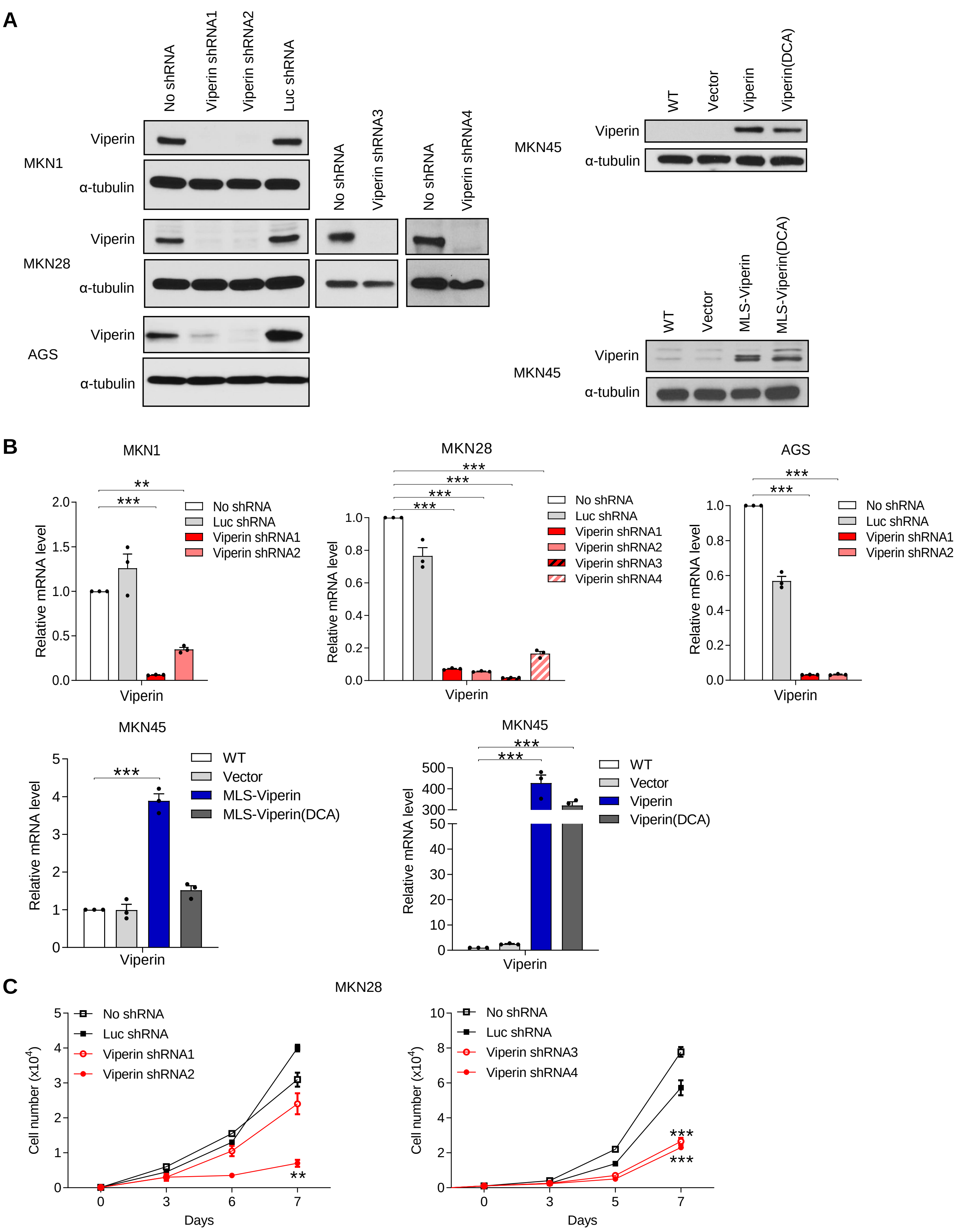

Supplemental Figure 3. Generation of viperin knockdown and stably expressing cancer cell lines. (**A** and **B**) No shRNA, a control luciferase (Luc) shRNA or Viperin shRNAs were stably expressed in MKN1, MKN28, and AGS. A control vector, viperin or viperin mutant (Viperin(DCA), MLS-Viperin or MLS-Viperin(DCA)) was stably expressed in MKN45. (**A**) Expression levels of viperin or viperin mutant in the stable cell lines were measured by immunoblot using MaP.VIP. α- tubulin was used as a loading control. (**B**) Relative mRNA levels of viperin or viperin mutant in the stable cell lines were measured by qRT-PCR and normalized to β-actin mRNA. Viperin(DCA), two cysteine residues (88 and 91) of viperin were mutated to alanine; MLS-Viperin or MLS-Viperin(DCA), the N-terminal amphipathic α-helix (residues 1 to 42) of Viperin or Viperin(DCA) was deleted and replaced by mitochondrial localization sequences (MLS, residues 2 to 34) of vMIA. Data are presented as means ± SEM (n = 3 in triplicate). (**C**) Proliferation of MKN28 viperin KD cells was measured. MKN28 stably expressing no shRNA, Luc shRNA or Viperin shRNAs were plated at 1×10^4 cells per well in 6-well plates and cultured at 37°C for 7 days. At the indicated times, the number of cells in triplicate wells were determined using the trypan blue exclusion method. Data are presented as means ± SEM (n = 3 in triplicate). Statistical analysis was performed by one-way ANOVA with Dunnett's multiple-comparison test (**B** and **C**). **$P < 0.01$; ***$P < 0.001$.

Supplemental Figure 4

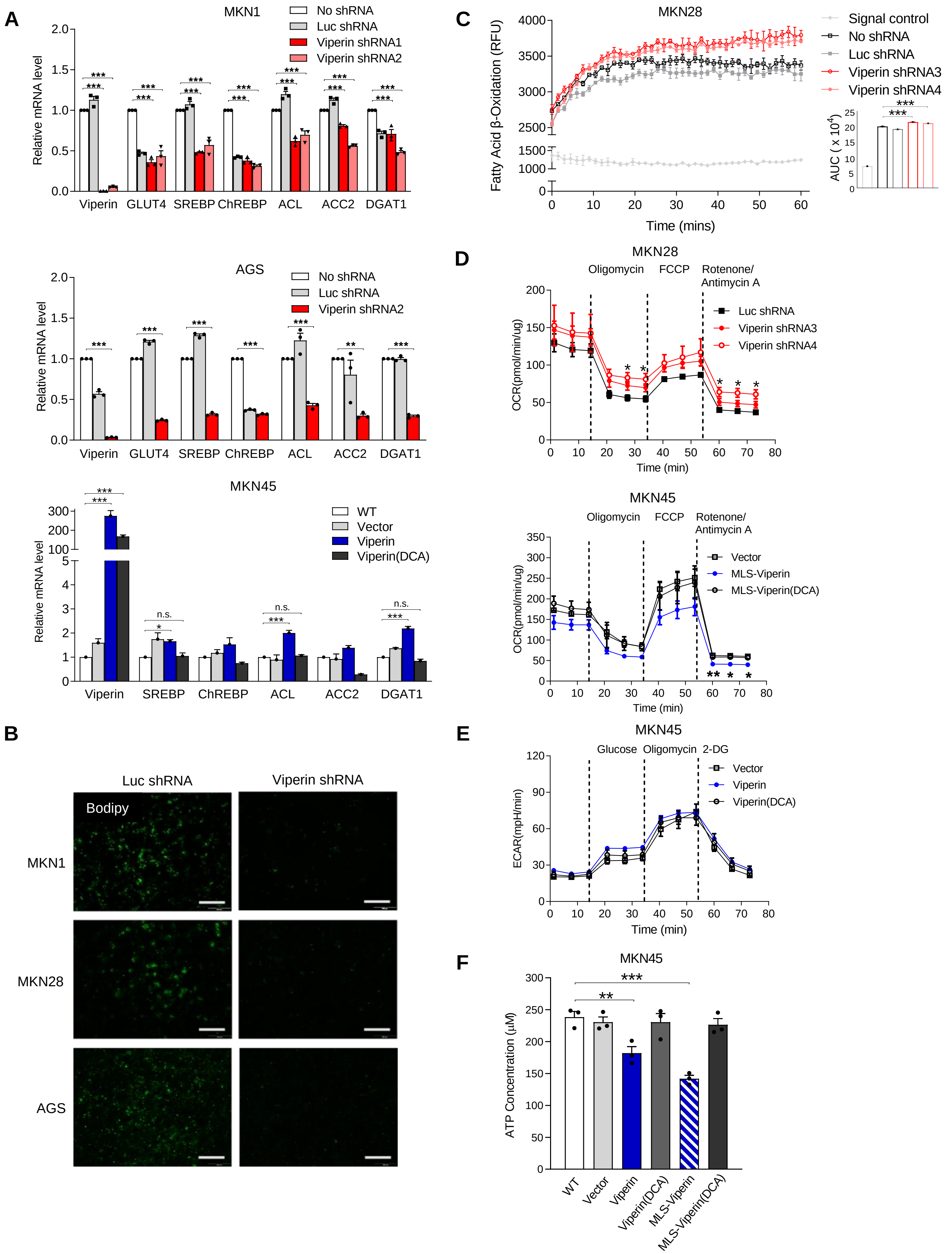

Supplemental Figure 4. Viperin-dependent metabolic alteration in cancer cells. (**A**) No shRNA, a control luciferase (Luc) shRNA or Viperin shRNAs were stably expressed in MKN1 and AGS, and a control vector, Viperin or Viperin(DCA) were stably expressed in MKN45. Relative mRNA levels of viperin, a glucose transporter (GLUT4), major transcriptional regulators (SREBP and ChREBP), and key lipogenic enzymes (ACL, ACC2 and DGAT1) in the stable cell lines were measured by qRT-PCR and normalized to β-actin mRNA. Data are presented as means ± SEM (n = 3 in triplicate). Viperin(DCA), two cysteine residues (88 and 91) of viperin were mutated to alanine. (**B**) Lipid levels in viperin KD cancer cell lines. MKN1, MKN28, and AGS stably expressing a control Luc shRNA or Viperin shRNA were stained with bodipy-FITC (green), an indicator for lipid droplets (LDs), Scale bar, 100 μm. Note that LDs were reduced in viperin KD cancer cell lines. (**C**) Fatty acid β-oxidation assay was performed for MKN28 stably expressing a control Luc shRNA or Viperin shRNAs. Fatty acid measurement medium without cells was used as a signal control. The area under the curve (AUC) was calculated and tested for significance. Data are presented as means ± SEM (n = 2 in triplicate). (**D**) OCR was measured in the MKN28 and MKN45 stable cell lines. Oligomycin, FCCP, rotenone, and antimycin A were added at the indicated time. Data are presented as means ± SEM (n = 3 in triplicate). (**E**) ECAR was measured in MKN45 stably expressing a control vector, Viperin or Viperin(DCA). Glucose, oligomycin, and 2-deoxyglucose (2-DG) were added at the indicated time. Data are presented as means ± SEM (n = 3 in triplicate). ***$P < 0.001$. (**F**) Cellular ATP levels were measured in MKN45 stable cell lines. A control vector, viperin or viperin mutant (Viperin(DCA), MLS-Viperin or MLS-Viperin(DCA)) was stably expressed in MKN45. Data are presented as means ± SEM (n = 2 in triplicate). Statistical analysis was performed by one-way ANOVA with Dunnett's multiple-comparison test (**A** and **C**-**F**). *$P < 0.05$; **$P < 0.01$; ***$P < 0.001$.

Supplemental Figure 5

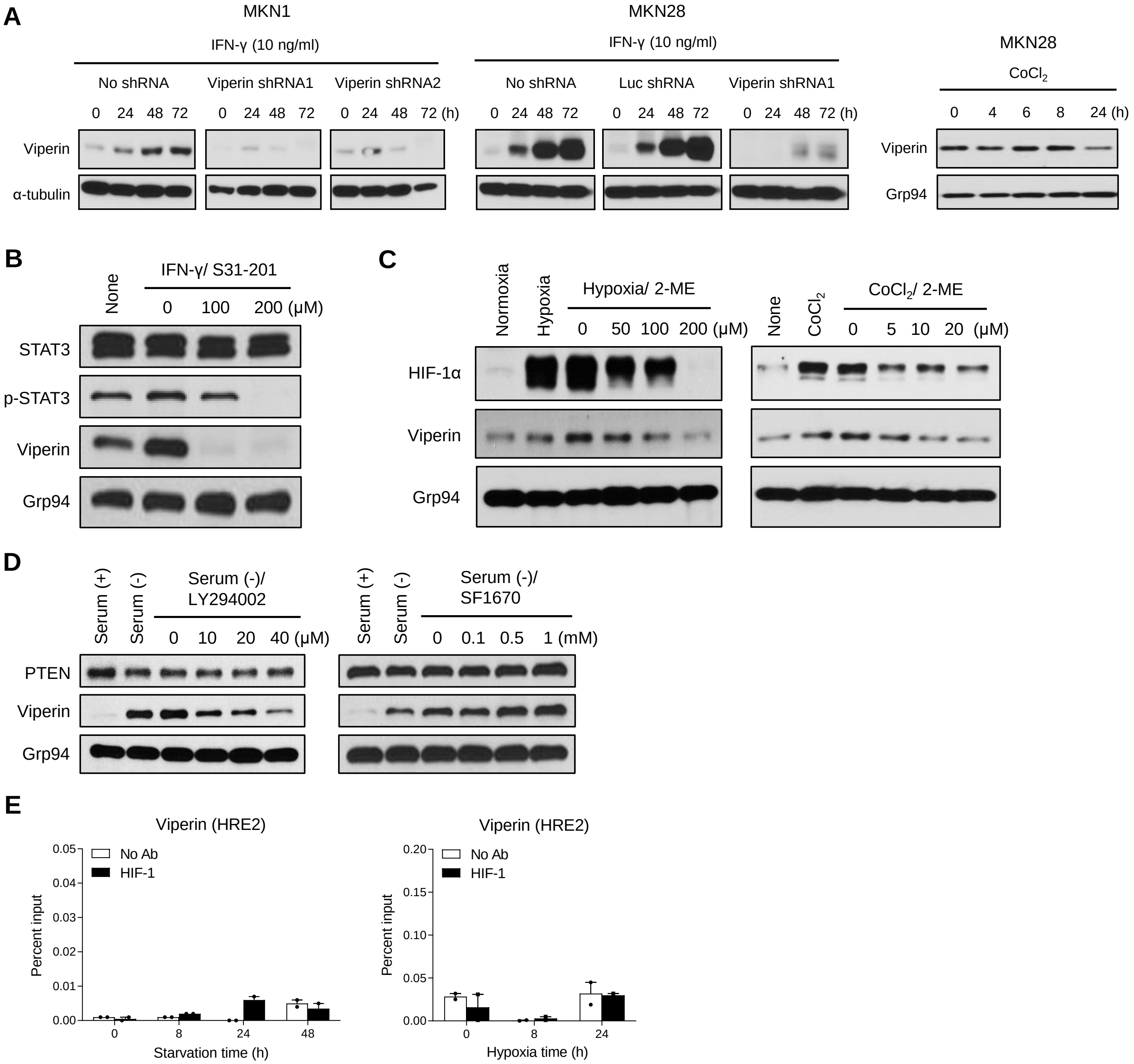

Supplemental Figure 5. Viperin is induced in the tumor microenvironment. (**A**) Viperin induction in MKN1 or MKN28 control and viperin KD cells treated with IFN-γ or the hypoxia-mimetic agent $CoCl_2$ for the indicated times. Viperin protein was detected by immunoblot using MaP.VIP. α- tubulin was used as a loading control. (**B**) MKN28 was treated with IFN-γ for 6 h and then added with S31-201, a STAT3 inhibitor at the indicated concentration for 24 h. Each protein was detected by immunoblot using specific monoclonal antibodies. Grp94 was used as a loading control. (**C**) MKN28 was cultured in hypoxia chamber for 24 h or treated with the hypoxia-mimetic agent CoCl2 for 8 h. The cells were treated with 2-ME at the indicated concentration. Each protein was detected by immunoblot using specific monoclonal antibodies. (**D**) MKN28 was cultured in the presence and absence of serum, and treated with LY294002 or SF1670 at the indicated concentration for 24 h. Each protein was detected by immunoblot using specific monoclonal antibodies. Grp94 was used as a loading control. LY294002, a PI3K/AKT inhibitor; SF1670, a PTEN inhibitor. (**E**) ChIP assay was performed for MKN28 cultured in serum-free media or hypoxia chamber for the indicated time. Chromatin samples were immunoprecipitated with a specific monoclonal antibody to HIF-1α and assessed by real-time PCR. Data are presented as means ± SEM (n = 2 in triplicate). Statistical analysis was performed by t test (**E**). Note that HIF-1α did not bind to HRE2 of viperin promoter under serum starvation or hypoxia.

Supplemental Figure 6

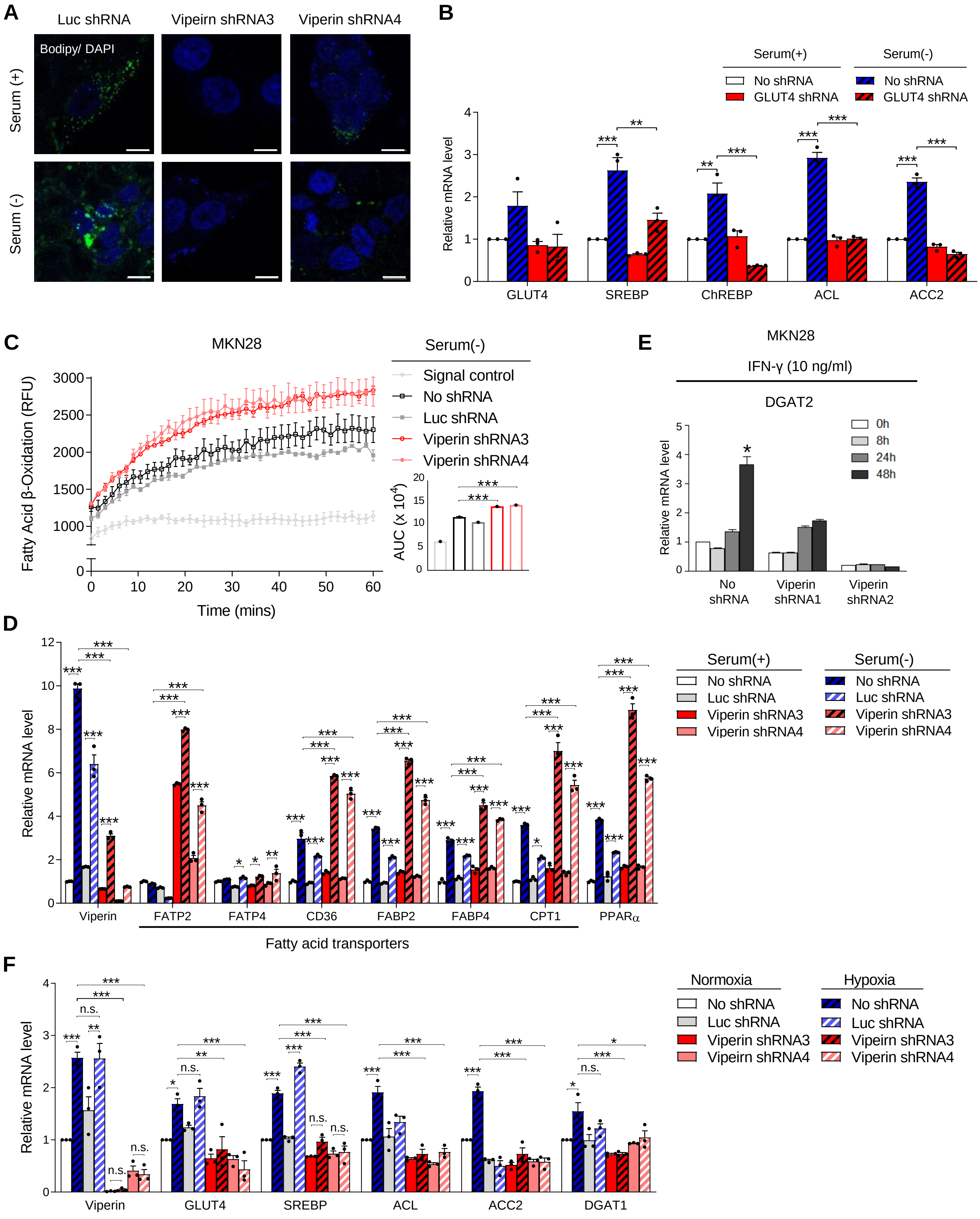

Supplemental Figure 6. Viperin modulates cancer metabolism in the tumor microenvironment. (**A**) MKN28 control and viperin KD cells were cultured in the presence and absence of serum for 48 h. The cells were stained with bodipy-FITC (green), an indicator for lipid droplets (LDs), Scale bar, 20 μm. Note that the accumulation of LDs were increased only in control cells in serum-free condition. (**B**) MKN28 control and GLUT4 KD cells were cultured in the presence and absence of serum for 48 h. Relative mRNA levels of the indicated genes in the stable cell lines were measured by qRT-PCR and normalized to β-actin mRNA. Data are presented as means ± SEM (*n* = 2 in triplicate). (**C** and **D**) MKN28 control and viperin KD cells were cultured in the presence and absence of serum for 48 h. (**C**) The level of fatty acid β-oxidation was measured in the stable cell lines. Fatty acid measurement medium without cells was used as a signal control. The area under the curve (AUC) was calculated and tested for significance. Data are presented as means ± SEM (*n* = 2 in triplicate). (**D**) Relative mRNA levels of viperin, fatty acid transporters (FATP2 and 4, CD36, FABP2 and 4, and CPT1), and a major transcriptional regulator (PPARα) in the stable cell lines were measured by qRT-PCR and normalized to β-actin mRNA. Data are presented as means ± SEM (*n* = 3 in triplicate). (**E** and **F**) MKN28 control and viperin KD cells were treated with IFN-γ for the indicated times (**E**) or cultured in normoxia or hypoxia chamber for 48 h at 37°C (**F**). Relative mRNA levels of the indicated genes in the stable cell lines were measured by qRT-PCR and normalized to β-actin mRNA. Data are presented as means ± SEM (*n* = 2 in triplicate). Statistical analysis was performed by one-way ANOVA with Dunnett's multiple-comparison test (C and E) or Tukey's multiple-comparison test (**B**, **D**, and **F**).*$P < 0.05$; **$P < 0.01$; ***$P < 0.001$.

Supplemental Figure 7

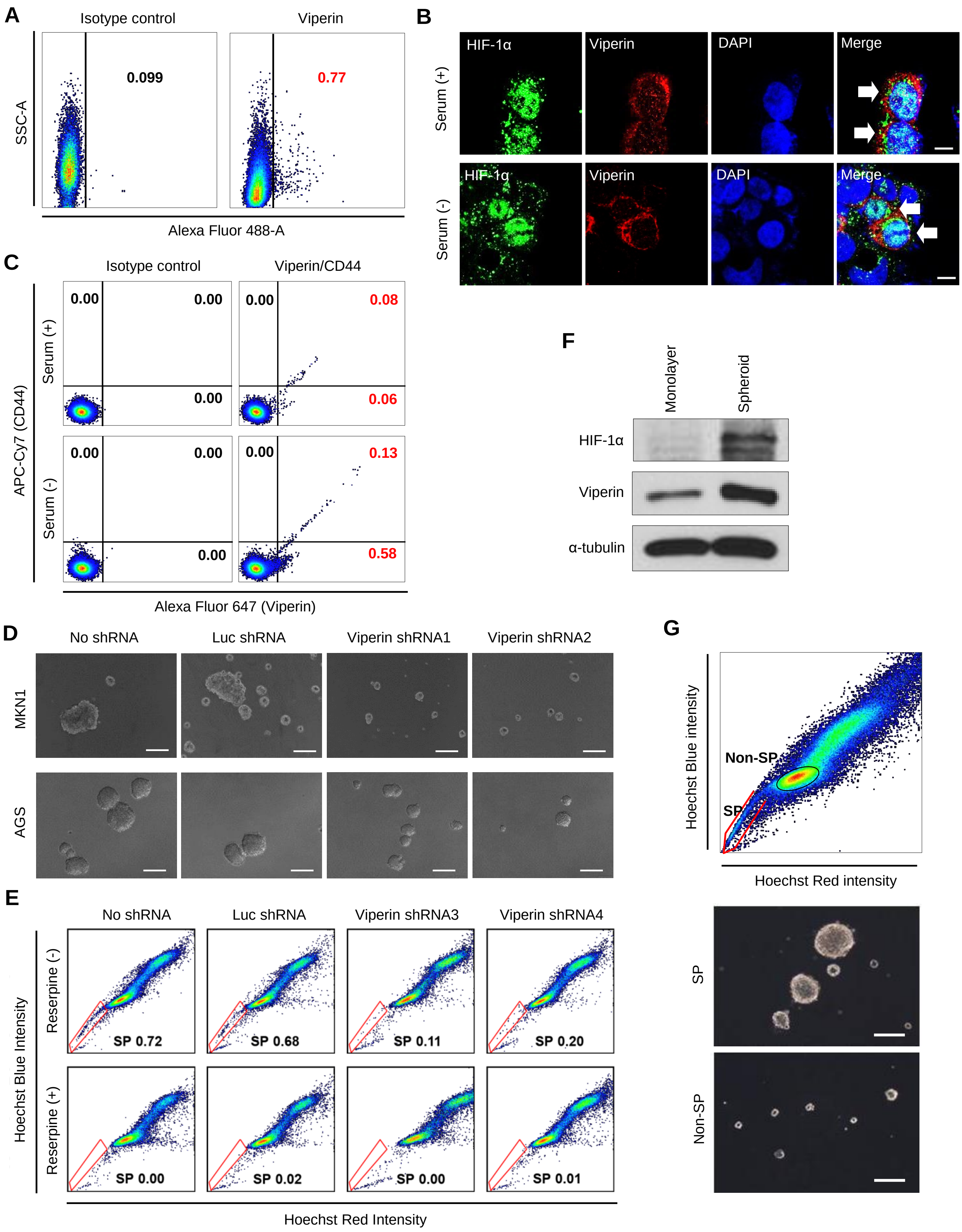

Supplemental Figure 7. Viperin is required to maintain CSC properties. (**A**) Flow cytometric analysis of viperin expressing cells in MKN28. Cells were stained with MaP.VIP, a primary antibody to viperin and an Alexa Fluor 488-conjugated secondary antibody. An isotype control antibody was used as a negative control. (**B**) Immunofluorescence staining for HIF-1α and viperin in MKN28 cultured in the presence and absence of serum for 48 h. The cells were stained with specific monoclonal antibodies to HIF-1α (green) and viperin (red). DAPI-stained nuclei (blue). The filled arrows indicate viperin expression in cells expressing HIF-1α. Scale bar, 20 μm. (**C**) Viperin expression in CSCs and non-CSCs under conditioned media. MKN28 cells cultured in the presence and absence of serum were stained with primary antibodies to CD44 and viperin, and APC-Cy7-conjugated and Alexa Fluor 647-conjugated secondary antibodies. CD44 expressing cells (APC-Cy7) and viperin expressing cells (Alex Fluor 647) were analyzed using flow cytometer. An isotype control antibody was used as a negative control. (**D**) Single cell-derived spheroid formation in the MKN1 and AGS stable cell lines. Spheroids were analyzed using an inverted microscope. A representative image of spheroid formation in the stable cell lines. Scale bar, 100 μm. (**E**) Analysis of side population (SP) in the MKN1 control and viperin KD cells. The stable cell lines were stained with Hoechst 33342 and analyzed using flow cytometer. Hoechst-unstained SP was gated in a red line and the percentage of SP was indicated. Reserpine-treated cells were used as a negative control for SP. (**F**) Expression of HIF-1α and viperin in MKN28 monolayers and spheroids. Each protein was detected by immunoblot using specific monoclonal antibodies. α-tubulin was used as a loading control. (**G**) Single cell-derived spheroid formation in the SP of MKN28. Cells were stained with Hoechst 33342 and were sorted to SP and non-SP cells. Spheroids were formed in SP and non-SP cells under conditioned media as described above. A representative image of spheroid formation in SP and non-SP cells. Scale bar, 100 μm.

Supplemental Figure 8

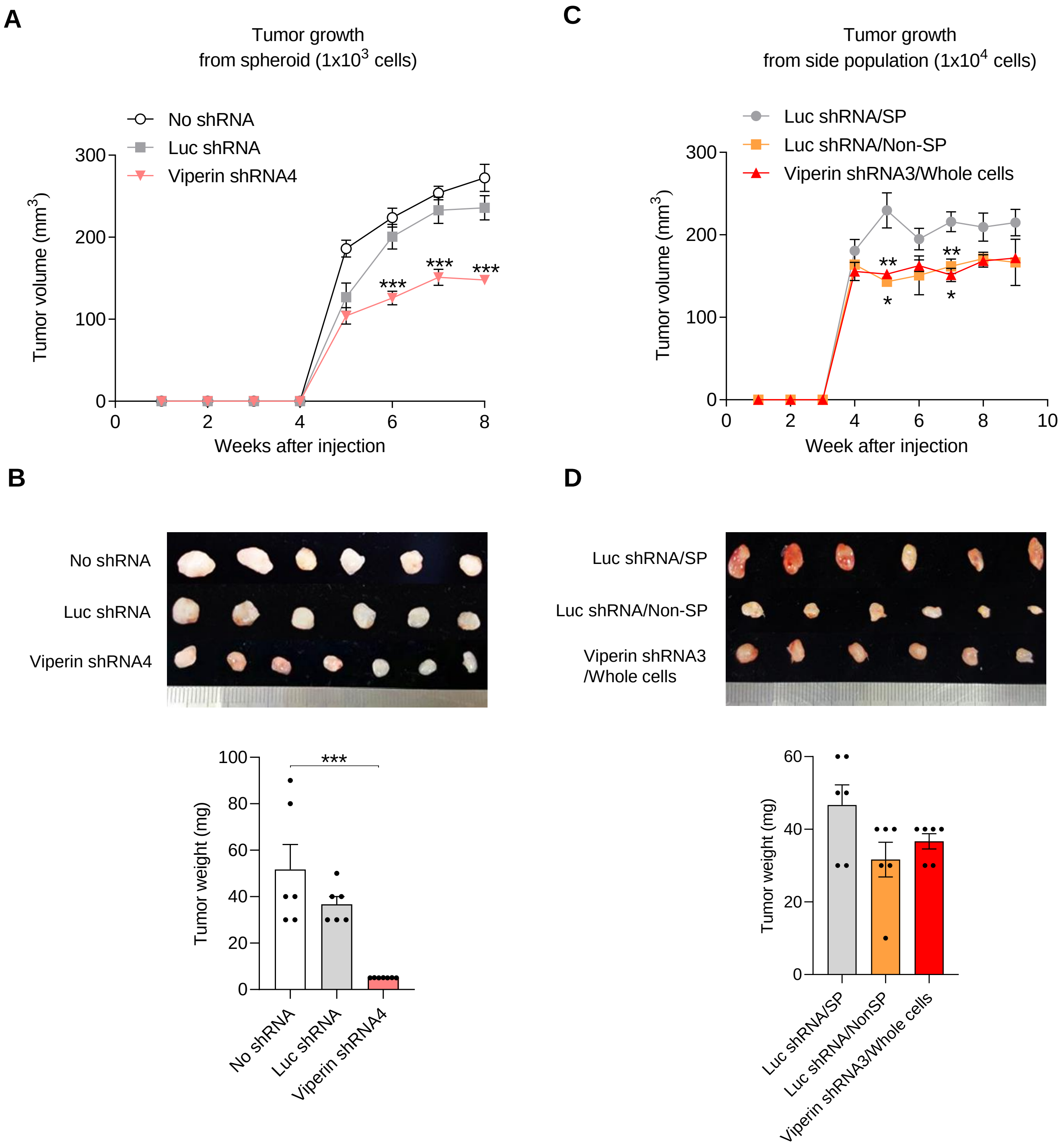

Supplemental Figure 8. Tumor growth in viperin knockdown cancer cell-derived xenograft mouse models. (**A** and **B**) Tumor growth in the MKN28 control and viperin KD cell-derived xenograft mouse models. (**A**) Spheroids of the stable cell lines were dissociated and counted. Single cell suspension was mixed with an equal volume of Matrigel. The mixture (1×10^3 cells/mouse) was injected subcutaneously into the flank of 6-week-old male nude mice (n = 6/cell line). Tumor growth was monitored weekly and tumor volume was measured using a metric caliper. (**B**) After 8 weeks, mice were sacrificed and tumors were isolated. Tumor size (top) and weight (bottom) were measured. Data are presented as means ± SEM (n = 6). (**C** and **D**) Tumor growth in SP and non-SP cell-derived xenograft mouse models. (**C**) MKN28 cells expressing Luc control shRNA were stained with Hoechst 33342 and were sorted to SP and non-SP cells using flow cytometry. Single cell suspension of SP cells or non-SP cells and viperin KD whole cells was mixed with an equal volume of Matrigel. The mixture (1×10^4 cells/mouse) was injected subcutaneously into the flank of 6-week-old male nude mice (n = 6/cell line). Tumor growth was monitored weekly and tumor volume was measured using a metric caliper. (**D**) After 10 weeks, mice were sacrificed and tumors were isolated. Tumor size (top) and weight (bottom) were measured. Data are presented as means ± SEM (n = 6). Statistical analysis was performed by one-way ANOVA with Dunnett's multiple-comparison test (**A**-**D**). *$P < 0.05$; **$P < 0.01$; ***$P < 0.001$.

Supplemental Figure 9

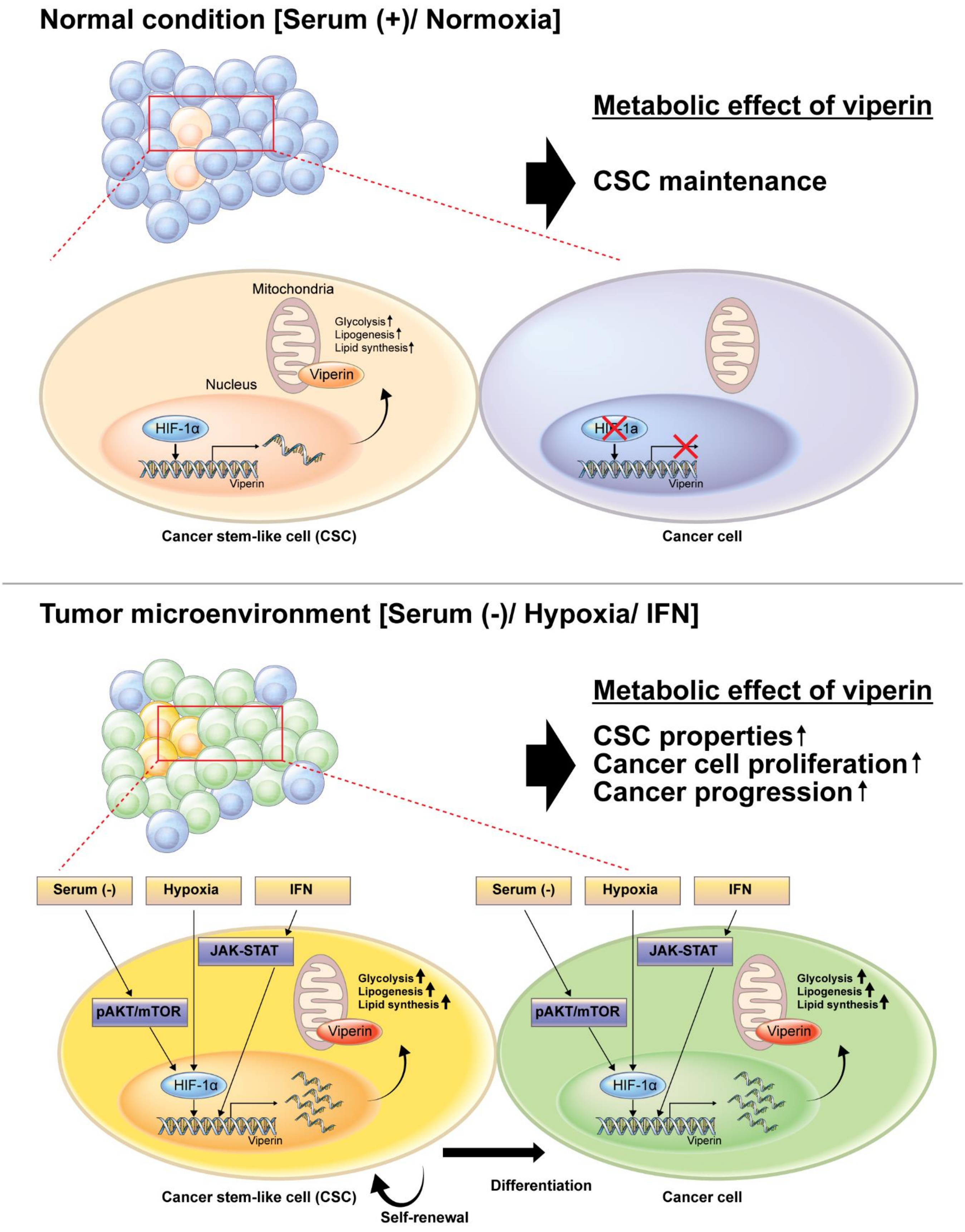

Supplemental Figure 9. Working model of viperin-dependent cancer metabolic reprogramming and cancer progression. In normal condition, viperin is expressed in CSCs through the HIF-1α pathway and induces glycolysis and lipid metabolism to support maintenance of their properties (**top**). In the tumor microenvironment such as serum depletion, hypoxia, and IFN secretion, viperin is induced in both CSCs and non-CSCs through PI3K/AKT/mTOR/HIF-1α pathway and JAK/STAT pathway, and promotes glycolysis and lipid metabolism to enhance CSC properties, cancer cell proliferation, and cancer progression (**bottom**).

Supplemental Table 1. Primer sequences used for qRT-PCR.

Primer name	Forward	Reverse
Viperin	TAGAGTCGCTTTCAAGATA	TTCAGATCAGCCTTACTCC
GLUT1	GCCCCTCAGTCGTTCTCAT	CCTTCTATTTGCGGTCCTCC
GLUT4	CTCAGCAGCGAGTGACTGG	AGCCACGTCTCATTGTAGCTC
SREBP	CGGAGCCATGGATTGCACT	TAGGCCAGGGAAGTCACTG
ChREBP	AGTGCTTGAGCCTGGCCTAC	TTGTTCAGGCGGATCTTGTC
ACL	TGTAACAGAGCCAGGAACCC	CTGTACCCCAGTGGCTGTTT
ACC2	GACCACAGGTGAAGCTGAGA	GTGTTCCCGTCCCCTCTTC
FAS	CATCCAGATAGGCCTCATAGAC	CTCCATGAAGTAGGAGTGGAA
DGAT1	TCGCCTGCAGGATTCTTTAT	GCATCACCACACACCAGTTC
DGAT2	TCACCTGGCTCAATAGGTCCA	CCAGCAATCAGTGCAGAATATG
Lgr5	TCTCGGTGAGCCTGAGAAAGC	ATGCTGGAGCTGGTAAAGGT
CD44	AGAGGCTGAGACAGGAGGTT	GCTTCCAGAGTTACGCCTT
ALDH	TTTGTCCAGCCCACAGTGTT	ACGCCATAGCAATTCACCCA
Nanog	TCTGGACACTGGCTGAATCC	TGACTGGATGGGCATCATGG
Sox2	TACAGCATGATGCAGGACCA	CGAGCTGGTCATGGAGTTGTA
Oct4	AGGTATTCAGCCAAACGACCA	GCACGAGGGTTTCTGCTTTG
FATP2	TGGTGTCGCCAGAACTACAAG	GAAAGAGTCAATCCCATCTGTGT
FATP4	CGGTTCTGGGACGATTGTAT	AACCTGGTGCTGGTTTTCTG
CD36	TCAAGTCCAGAAGGGCGTGC	GCTTGGGCTCAAGGGTAGTGG
FABP2	ATTTCCATTCATGCCAAAG	TCCACTACATTCCAGCCTGA
FABP4	ACCTTAGATGGGGGTGTCCT	ACGCATTCCACCACCAGTTT
CPT1	CAGGAAGTTGCACCCTGGCA	ACTACACTCCAGCCTCGGCA
PPARα	AAGAGGTCGGACATGGGCCT	AGTGTGGTGGCGTGACCTTG
β-actin	GCTCCGGCATGTGCAA	AGGATCTTCATGAGGTAGT

Supplemental Table 2. Primer sequences used for ChIP assay.

Primer name	Forward	Reverse
Viperin (HRE1)	ACTGCTGTTGGGATGCTTTT	CCTGTATGCCAGGCAACTCT
Viperin (HRE2)	CGTCTTGCACCTCCCAGAG	CCTGGATGACCCTGACACC
FOXM1	GCCATTCTATCTTCAGGGCC	CGCGAAACCTGTCTTTTGCC
Untr12	TCCCTCCTGTGCTTCTCAG	AATGAACGTGTCTCCCAGAA
